# Genetic Control and Comparative Genomic Analysis of Flowering Time in Setaria (Poaceae)

**DOI:** 10.1534/g3.112.005207

**Published:** 2013-02-01

**Authors:** Margarita Mauro-Herrera, Xuewen Wang, Hugues Barbier, Thomas P. Brutnell, Katrien M. Devos, Andrew N. Doust

**Affiliations:** * Department of Botany, Oklahoma State University, Stillwater, Oklahoma 74078; † Department of Crop and Soil Sciences, and Department of Plant Biology, University of Georgia, Athens, Georgia 30602; ‡ China Tobacco Gene Research Center, High-Tech Zone, Zhengzhou, People’s Republic of China, 450001; § Boyce Thompson Research Institute, Cornell, Ithaca, New York 14853; ** Donald Danforth Plant Science Center, St. Louis, Missouri 63132

**Keywords:** *Setaria*, foxtail millet, QTL mapping, flowering time, comparative genomics

## Abstract

We report the first study on the genetic control of flowering in Setaria, a panicoid grass closely related to switchgrass, and in the same subfamily as maize and sorghum. A recombinant inbred line mapping population derived from a cross between domesticated *Setaria italica* (foxtail millet) and its wild relative *Setaria viridis* (green millet), was grown in eight trials with varying environmental conditions to identify a small number of quantitative trait loci (QTL) that control differences in flowering time. Many of the QTL across trials colocalize, suggesting that the genetic control of flowering in Setaria is robust across a range of photoperiod and other environmental factors. A detailed comparison of QTL for flowering in Setaria, sorghum, and maize indicates that several of the major QTL regions identified in maize and sorghum are syntenic orthologs with Setaria QTL, although the maize large effect QTL on chromosome 10 is not. Several Setaria QTL intervals had multiple LOD peaks and were composed of multiple syntenic blocks, suggesting that observed QTL represent multiple tightly linked loci. Candidate genes from flowering time pathways identified in rice and Arabidopsis were identified in Setaria QTL intervals, including those involved in the *CONSTANS* photoperiod pathway. However, only three of the approximately seven genes cloned for flowering time in maize colocalized with Setaria QTL. This suggests that variation in flowering time in separate grass lineages is controlled by a combination of conserved and lineage specific genes.

Flowering is a major developmental transition in the life-history of plants, and the genetic manipulation of flowering time has been crucial in the domestication and spread of cereal grasses such as wheat, rice, and maize. However, much of our knowledge on the genetic control of flowering in grasses is derived from studies in rice, and it is unclear to what extent flowering time pathways are shared across grasses, or what the relative importance of the separate pathways are in the different grass subfamilies. Our understanding of the genetics of flowering time is particularly poor in the subfamily Panicoideae, containing sorghum and maize, where relatively few genes controlling flowering have been identified. In addition to sorghum and maize, in the tribe Andropogoneae, a third species of panicoid grass, foxtail millet (*Setaria italica*), in the tribe Paniceae, recently has been sequenced (Bennetzen *et al.* 2012). The availability of a recombinant inbred mapping population and a dense genetic map from a cross between foxtail millet and its wild relative green millet (*Setaria viridis*) gives an opportunity to examine the genetic control of flowering in Setaria. Furthermore, the availability of the genome sequence allows us to examine the extent to which there is conservation of genetic and genomic architecture for this trait across panicoid grasses.

Much of what is known about control of flowering is derived from studies in Arabidopsis, rice and the pooid grasses wheat, barley, and Brachypodium (Higgins *et al.* 2010). In Arabidopsis, both autonomous and environmentally mediated flowering time pathways have been identified (Amasino 2010). These pathways act in mature leaves and converge on a central integrating protein, *FLOWERING LOCUS T* (*FT*), which is transported to the apical meristem to promote transition of the vegetative meristem to an inflorescence meristem (Corbesier *et al.* 2007). Photoperiod and vernalization genetic pathways allow Arabidopsis to adjust flowering time responses across its geographic range. For example, in most temperate regions, Arabidopsis is a winter annual, germinating in the fall, overwintering, and then being stimulated to flower by lengthening days in the spring. Plants that germinate in the summer and fall are prevented from flowering by the repression of FT by *FLOWERING LOCUS C* (*FLC*), under regulation by *FRIGIDA* (*FRI*) (Shindo *et al.* 2005). Vernalization over the winter reduces the sensitivity of *FLC* to *FRI*, turning off *FLC* expression, and releasing the floral mobile signal FT from suppression (Amasino 2010). FT expression is amplified by up-regulation of *CONSTANS* (*CO*) in the photoperiod pathway as a result of the increasing day-length of spring (Yanovsky and Kay 2002).

The grasses also possess multiple pathways to control flowering time, only some of which are conserved with Arabidopsis. One conserved pathway is the CONSTANS (CO) photoperiod pathway, found in all terrestrial plants and in algae (Serrano *et al.* 2009; Valverde 2011). However, the regulation of the genes in this pathway has diverged over time. For instance, *CO* acts as a positive regulator of *FT* under long day conditions in winter annuals such as Arabidopsis, winter wheat, and barley (Greenup *et al.* 2009), whereas in the same conditions in rice (a short day plant) the ortholog of *CO*, *HEADING DATE 1* (*HD1*), acts to suppress the *FT* ortholog *HEADING DATE 3A* (*HD3A*) (Izawa *et al.* 2002; Hayama *et al.* 2003; Song *et al.* 2010).

Rice also possesses a separate photoperiod regulated genetic pathway centered on *EARLY HEADING DATE 1* (*EHD1*), which acts with *HD1* to promote flowering via both *HD3A* and its co-ortholog *RICE FLOWERING LOCUS T 1* (*RFT1*) in short day environments, but which acts alone on *RFT1* to promote flowering under long day conditions (Komiya *et al.* 2009). *EHD1* is negatively regulated by *GRAIN HEADING DATE7* (*GHD7*) in long day conditions, and natural variation in *GHD7* has been shown to regulate the local adaptation of rice cultivars to different latitudes (Xue *et al.* 2008).

The *FLC-FRI* vernalization pathway is not found in monocots, although winter annual species in the Pooid subfamily, such as wheat, barley, rye, and Brachypodium have an analogous genetic pathway involving *VERNALIZATION1* (*VRN1*) and *VERNALIZATION2* (*VRN2*) (Yan *et al.* 2003, 2004). Pooid grasses are long day plants, where vernalization up-regulates *VRN1* expression, down-regulating *VRN2*, and removing the suppressive effect of *VRN2* on the *FT* ortholog *VERNALIZATION3* (*VRN3*) (Yan *et al.* 2006; Trevaskis *et al.* 2007). However, the vernalization pathway has not been described for rice, maize, sorghum, and the millets, which are either from tropical regions (rice, sorghum, maize) or are spring or summer annuals (foxtail millet).

Flowering time pathways in the grasses have been characterized in rice and the pooid grasses (Higgins *et al.* 2010) but are less well understood in the panicoid grasses. A few genes underlying variation in flowering time have been cloned in maize and sorghum, including *INDETERMINATE SPIKELET1* (Colasanti *et al.* 2006), the noncoding control region of ZmRap2.7, VEG*ETATIVE TO GENERATIVE TRANSITION1* (VGT1) (Salvi *et al.* 2007), *DWARF 8* (Thornsberry *et al.* 2001; Camus-Kulandaivelu *et al.* 2006), *ZmCCT*, a homolog of the rice photoperiod pathway gene *GHD7* (Hung *et al.* 2012), *CONZ1*, a homolog of *CO* (Miller *et al.* 2008), *ZFL1* and *ZFL2*, homologs of *LEAFY* in Arabidopsis (Bomblies *et al.* 2003), and *DELAYED FLOWERING1* (*DLF1*), which is an ortholog of *FLOWERING LOCUS D* (*FD*) (Muszynski *et al.* 2006). In sorghum *PSEUDO RESPONSE REGULATOR37* (*PRR37*) has been identified as the gene underlying *Ma1*, the locus that has the largest effect on flowering time and inflorescence maturation in sorghum (Murphy *et al.* 2011). In addition, quantitative genetic analyses have found four to six major quantitative trait loci (QTL) regions controlling flowering time variation in maize (Chardon *et al.* 2004; Salvi *et al.* 2009; Coles *et al.* 2010, 2011; Wang *et al.* 2010; Xu *et al.* 2012). There are also likely a large number of QTL of small effect that control flowering time, with evidence for allelic series at most loci (Buckler *et al.* 2009). In sorghum, a short day tropical species, meta-analysis of multiple QTL trials projected against a dense single-nucleotide polymorphism (SNP) map, suggests up to 17 loci affecting flowering time (Mace and Jordan 2011).

Sorghum and maize are panicoid crops that were domesticated in short-day environments, but foxtail millet (*Setaria italica*) was most likely domesticated from green millet (*S. viridis*) in the northern part of China, with more pronounced seasonal changes in photoperiod (Li and Wu 1996; Bettinger *et al.* 2010). Green millet is of interest in its own right, as it is a world-wide weed, adapted to multiple photoperiod regimes, including both short- and long-day cycles (Holm 1997; Dekker 2003), and a model for biofuels genetics, C4 photosynthesis research, and plant architectural modeling (Doust *et al.* 2009; Li and Brutnell 2011). A Sanger (Bennetzen *et al.* 2012) and Illumina (Zhang *et al.* 2012) genome sequence recently have been completed, along with several green millet accessions (Bennetzen *et al.* 2012). As part of the Sanger genome assembly effort an F7 recombinant inbred line (RIL) population of a cross between foxtail and green millet was genotyped using SNP markers, resulting in a 1000-loci genetic map (Bennetzen *et al.* 2012). We have used this population to investigate the genetic control of flowering time between foxtail and green millet in a variety of environments, to suggest candidate genes controlling flowering time variation in this population, and to compare QTL regions with those in the other domesticated panicoid grasses, maize and sorghum.

## Materials and Methods

### Plant materials, experimental design, and phenotyping

A total of 182 F7 RILs from an interspecific cross between *S. italica* accession B100 × *S. viridis* accession A10 (Wang *et al.* 1998; Bennetzen *et al.* 2012) were evaluated for flowering time in eight different trials. Two of the eight trials were conducted in greenhouses (GH) at Oklahoma State University (OK), four were conducted in the field (F) at Oklahoma State University and the University of Georgia (GA), and two were conducted in growth chambers (GC) at Oklahoma State University and the Boyce Thompson Institute, Ithaca, NY (BT). For the field trials, seed was germinated in greenhouses and transplanted into the field at the two- or three-leaf stage. The trials varied in photoperiod, temperature, plant spacing, and other environmental variables, with the two growth chamber experiments representing the shortest light period of 12 hr, the field trials and one greenhouse trial having light periods from 13 to 14.5 hr, and one greenhouse trial having a light period of 16 hr (Table 1). In addition, a growth chamber trial was performed in which the *S. viridis* A10 and *S. italica* B100 parents were grown under 12- and 16-hr light conditions, in the absence of other environmental variation, to examine the effect of photoperiod (see Supporting Information, File S1, for a comprehensive description of plant preparation and growing conditions).

**Table 1:** Summary of growth conditions

Trial	Mean Day Length, Hours	Mean Light Intensity, µmol.m^-2^.s^-1^	Average Max. Temperature, °	No. RILs Used	Comments
GH1-OK	14	1400	26	182	
GH2-OK	16	1400	26.5	107	
F1-OK	14.2	2200	26.5	182	
F2-OK	14.2	2200	28	182	
F1-GA	14.3	2200	28	182	Seeds vernalized after planting Seeds vernalized after planting
F2-GA	14.3	2200	28	182	
GC-BT	12	750	31	182	
GC-OK	12	350	28	126	

GH, greenhouse; OK, Oklahoma State University, Stillwater, OK; F, field; GA, University of Georgia, Athens, Georgia; GC, growth chamber; BT, Boyce Thompson Institute, Ithaca, New York.

### Phenotypic measurement

Our primary interest is in understanding the genetic regulation of commitment to flowering, which is when the shoot apical meristem becomes an inflorescence meristem. We used the number of days until the inflorescence on the main culm was first visible in the sheath of the flag leaf (days to heading) as the measurement of time to flowering, for its reliability and ease of measurement. We did not measure time to anthesis or time to stigma exsertion (‘silking’) because Setaria species have inflorescences with multiple orders of branching, and flowers on the different branches and in different parts of the inflorescence open at different times (Doust and Kellogg 2002).

### Molecular marker development

Most of the markers used in the QTL analysis map were SNP markers genotyped on an Illumina Golden Gate array, with SNP identification and probe design based on next-generation sequence data obtained in a random set of 48 of the RILs (Bennetzen *et al.* 2012). We added a number of simple sequence repeat (SSR), sequence-tagged-site (STS), and gene markers to the map, in order to compare the RIL map with previously published maps (Wang *et al.* 1998; Jia *et al.* 2009).

Almost 200 published SSR primer pairs (Jia *et al.* 2007, 2009; Gupta *et al.* 2012) were tested on the parents, and a total of 126 informative markers were chosen to complement the SNP markers. Polymerase chain reaction (PCR) fragment separation for SSR markers was done via agarose gels (1−3% depending on fragment sizes) or with an ABI PRISM 3730 Genetic Analyzer (Applied Biosystems). We also developed several STS markers for selected rice RFLP probes/sequences used in the F_2_ foxtail genetic map (Wang *et al.* 1998) and for some genes of interest (Table S1). Most of these were detected using tetra-prime ARMS-PCR, a method whereby two primer pairs can be used to amplify the two different alleles of a SNP in the same PCR (Ye *et al.* 2001) (see File S1 for details of molecular marker development and genotyping; see File S2 for genotypic and phenotypic scores). The rest were detected using enzymes that cut one or other allelic copy.

### Genetic map development

Marker linkage analysis was done with the program JoinMap 4 (Van Ooijen 2006). Molecular markers were grouped using a maximum recombination frequency of 0.25 and a LOD (logarithm of odds ratio) threshold of 4, 5, or 6 (in one instance the use of a LOD threshold of 6 was necessary to separate linkage groups). Marker ordering calculation was done with the maximum likelihood mapping algorithm. The Kosambi function was used to convert recombination frequencies to cM distances. A single, most informative marker was selected from clusters of markers with the purpose to improve QTL mapping. Suspicious genotypic scores identified from the genotype probabilities table calculated by JoinMap, were verified and edited when necessary.

### QTL analyses

SPSS version 19 (Armonk, NY) was used to calculate least square means, and QTL Cartographer Unix version 1.16 (Basten *et al.* 1994, 2002) and WinQTLCart version 2.5 (Wang *et al.* 2011) were used to perform the QTL analyses. QTL mapping was done with the composite interval mapping (CIM) method, using a genome scan interval of 1 cM, a window size of 10 and the forward and backward regression method (Jansen and Stam 1994; Zeng 1994). LOD threshold values were estimated via 1000 permutations (Churchill and Doerge 1994; Doerge and Churchill 1996).

### Epistasis

Epistatic interactions were calculated with the program Epistacy (Holland 1998). Epistasis was measured for all pairs of markers, giving 233,586 tests for significance. Correcting for linked markers, using the eigenvalue variance method (Cheverud 2001), gives 222,826 tests, and a Bonferroni adjustment to the experiment-wide error rate of *P* < 0.05 gave an individual test P-value of *P* < 2.3 × 10^−7^ (Lynch and Walsh 1998).

### Comparative genomic and candidate gene analysis

To compare QTL regions among the panicoid grasses, genomic coordinates of each Setaria QTL region were identified using colocalized markers from the genetic map (Bennetzen *et al.* 2012). Where QTL from multiple trials colocalized in the same map positions, common regions were defined as the region of overlap between individual QTL intervals (Figure 2). Syntenic dotplots were generated for comparisons between Setaria and sorghum and between Setaria and maize using the Synmap module in CoGe (Lyons and Freeling 2008; Tang and Lyons 2012). Setaria genome version 2.1 (id12240), sorghum genome version 1.4 (id93), and maize genome version 2 (id333) were used. We configured CoGe to assign gene pairs to classes based on their Ks values and thus to distinguish orthologous syntenic regions (resulting from speciation from a common ancestor) from paralogous syntenic regions that were the product of the much older pan-grass whole genome duplication (Paterson *et al.* 2004; Schnable and Lyons 2011). Only orthologous syntenic regions were used to compare QTL, and we use the term syntenic to refer to such regions in the rest of this paper. Syntenic depth was set as 1:1 between the diploid species sorghum and Setaria and 2:1 for maize and Setaria because of the additional whole genome duplication in maize (Blanc and Wolfe 2004; Swigonova *et al.* 2004). Syntenic regions in maize and sorghum were scanned for published maize and sorghum flowering time QTL (Buckler *et al.* 2009; Coles *et al.* 2010; Mace and Jordan 2011).

Two approaches were used to identify candidate genes. First, more than 100 genes for flowering time were identified from the literature, using recent reviews (Greenup *et al.* 2009; Amasino 2010; Higgins *et al.* 2010; Song *et al.* 2010). Protein and nucleotide sequences of the flowering time genes were used in BLAST searches against *S. italica* genome sequence version 2.1 (www.Phytozome.net). The location of candidate genes on the foxtail millet genomic sequence was then compared to that of the QTL regions. Top hits in QTL regions were used in a reciprocal BLAST search to confirm identical query-hit pairs to flowering time genes. The second approach analyzed nucleotide and peptide sequences of all genes identified within each QTL interval. These were used as queries in BLASTP searches against the SwissProt database (Uniprot-Consortium 2012), and then Blast2GO (Conesa *et al.* 2005) was used to obtain gene ontology terms. These gene ontology terms were searched using “flower*” and related terms and candidate genes identified in this way were used as input to HMMer3.0 (Finn *et al.* 2011). HMMer 3.0 was used to annotate the genes in the Pfam database (Punta *et al.* 2012), and HMMer output was manually curated and traced back to the original publication to validate gene information. A gene was chosen as a real QTL candidate only if there was experimental evidence that it affected flowering. The presence of syntenic orthologs of candidate genes across the three genomes was also investigated.

## Results

### Phenotypic variation

**Figure 1:**
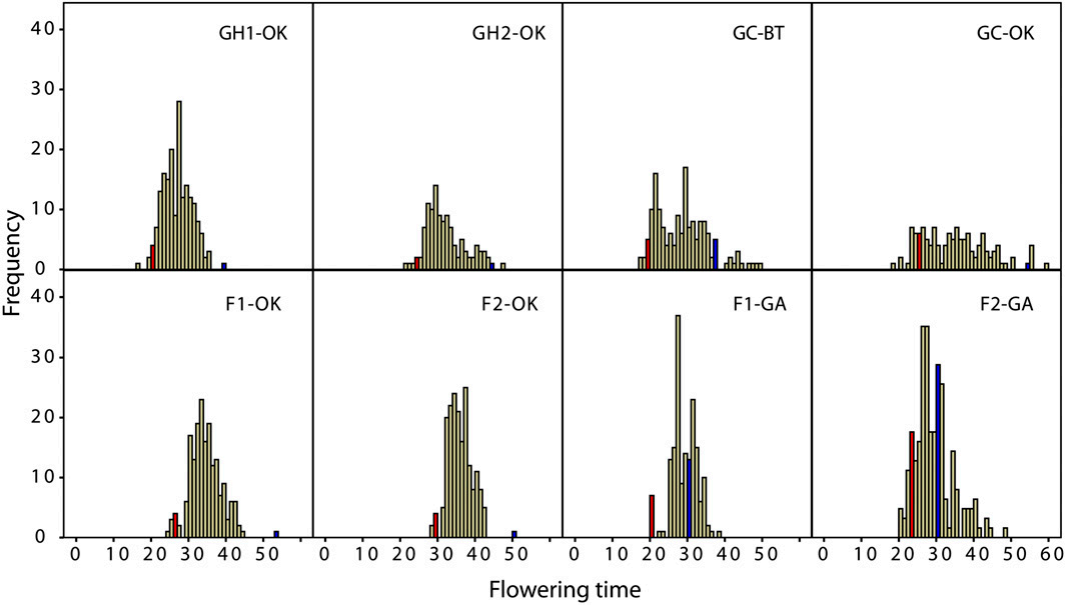
Histograms of the distribution of flowering times for each trial. The flowering time of the *S. viridis* parent is indicated by a red column and that of the *S. italica* parent by a blue column. GH, greenhouse; GC, growth chamber; BT, Boyce Thompson Institute, Ithaca, NY; OK, Oklahoma State University, Stillwater, OK; F, field; GA, University of Georgia, Athens, GA.

There was substantial variation in flowering time among the eight trials (Figure 1). The green millet parent always flowered earlier than the foxtail millet parent, and in all trials it was amongst the earliest accessions to flower (Figure 1). The foxtail millet parent was more variable in flowering time compared to the RILs, although in most trials it was among the latest to flower. Most trials showed at least some evidence of transgressive segregation, with RILs flowering both earlier than green millet and later than foxtail millet, although in only three trials was there a large amount of transgressive segregation. Growth trials of the parents under 12-hr and 16-hr photoperiod regimes in a constant growth chamber environment confirmed that *S. viridis* flowers earlier than *S. italica*, and that both species flower later and produce more leaves under the 16-hr than under the 12-hr light period (Table 2).

**Table 2:** Mean days to flowering and mean leaf number at flowering for the parental accessions of *S. viridis* A10 and *S. italica* B100 under 12- or 16-hr light conditions

	*S. viridis* (12 hr)	*S. italica* (12 hr)	*S. viridis* (16 hr)	*S. italica* (16 hr)
Days to flowering	24.4 (0.39)	52.5 (0.63)	31.4 (0.27)	74.2 (1.84)
Leaf number	7.0 (0.0)	17.9 (0.35)	11.6 (0.17)	24.9 (0.60)

Values in parentheses represent the standard error of each estimate.

The distribution of flowering time differed between trials, with six of the eight trials showing a “bell”-shaped distribution and two showing either a flat or apparent bimodal distribution. The single trial with a relatively flat distribution (GC-OK) can be explained by the *a priori* selection of genotypes to represent the ends of the distribution, in order to have the most power possible in a trial where space limitations meant that only a restricted number of plants could be grown. The apparent bimodal distribution in GC-BT is mirrored to some extent in F1-GA and F2-GA, and suggests that there are substantial RIL by environment interactions. An analysis of variance (not shown) with trial and RIL as random factors showed significant differences in both main effects and the interaction between trial and RIL.

### Genetic map

A total of 182 RILs were used to construct a genetic map, covering a total of 1125.4 cM. The map uses 684 uniquely positioned markers (only the most informative marker was selected from clusters of cosegregating markers), and includes 560 SNP markers, 101 SSR markers, six STS markers developed from specific genes, and 17 STS markers developed from RFLP markers used in the original F2 map (Wang *et al.* 1998). The six genes successfully genotyped were *teosinte branched1* (*tb1*), *barren stalk1* (*ba1*), a gene homologous to *barren inflorescence2* (*bif2-like*), *dwarf3* (*d3*), *MORE AXILLARY BRANCHES 1* (*MAX1*), and *MONOCULM 1* (*MOC1*). Genbank accessions for these markers are in File S1.

Marker order was well conserved for the SNP markers with the published foxtail millet map (Bennetzen *et al.* 2012). Sixty of the 70 SSR markers previously mapped in the F2 population mapped to similar positions in our map, and 10 mapped to a different position but to the correct linkage group (Jia *et al.* 2009). In addition, we had 17 shared markers with the original F2 map (Wang *et al.* 1998) and these were ordered in the same way in both maps. There were seven regions with gaps bigger than 10 cM, in linkage groups I, III (two gaps), V, VI, VII, and IX. Distance between markers ranged from 0.1 to 16.9 cM; the average distance between markers was 0.82 cM. Linkage group length ranged between 94.2 cM (VI) and 182.7 cM (IX). As previously reported (Bennetzen *et al.* 2012), significant segregation distortion was observed on most of linkage group II, and several sections of linkage groups III, IV, V, VII, and IX, because of an excess of the *S. italica* alleles in those regions. A 50-cM section on top of linkage group VI and a 62 cM section at the bottom of linkage group VII showed distorted segregation ratios too but in this case the *S. viridis* homozygotes were favored.

### QTL analyses

**Figure 2:**
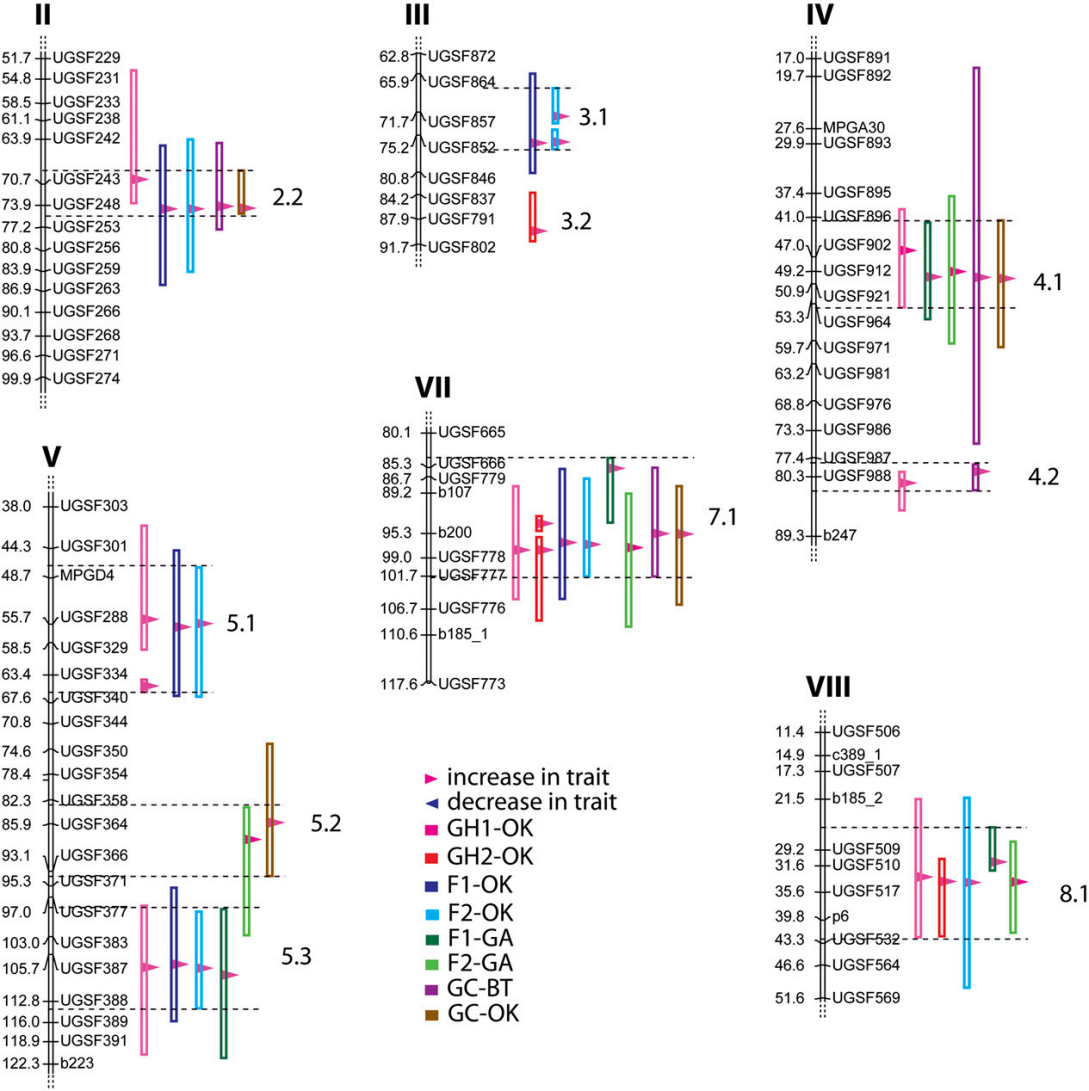
Major QTL regions found, showing QTL intervals from each trial and common regions searched for candidate genes. Dashed black lines delimit common QTL regions defined by overlapping QTL intervals. A full version of this figure is represented in Figure S1 and LOD curves for all trials in Figure S2.

A total of 16 flowering time QTL were identified in the eight trials, with nine identified in at least two trials (Figure 2, Figure S1, Figure S2, Table 3). The percentage of phenotypic variance explained by individual QTL ranged between 2.5% (for QTL 6.1 and 9.1) and 41.9% (QTL 4.1 in the GC-OK trial), and the total percentage of phenotypic variation explained by flowering time QTL on a per trial basis ranged from a low of 34.7% for the F1-GA trial to 88.1% for the GC-BT trial (Table 3). Of the nine QTL consistently identified in at least two trials, the one on linkage group IV (QTL 4.1) had the highest average percentage of phenotypic variation explained (23.84%), and was identified in five trials. Several QTL, such as QTL 4.1, 5.1, and 8.1, had multiple LOD peaks along the QTL interval, suggesting that several tightly linked loci may underlie a single QTL region.

**Table 3:** Additive effects in days and percentage of variation explained (in parentheses) for QTL identified in the eight trials

Linkage group	QTL	GH1-OK	GH2-OK	F1-OK	F2-OK	F1-GA	F2-GA	GC-BT	GC-OK
I	1.1	0.9 (6.0)							
II	2.1						−1.2 (4.0)		
	2.2	1.6 (15.6)		1.5 (11.3)	1.2 (14.4)			2.6 (12.0)	2.1 (3.7)
III	3.1			1.0 (6.7)	0.6 (3.3)				
	3.2		1.7 (10.7)						
IV	4.1	0.9 (5.9)				1.0 (8.7)	2.5 (22.9)	4.6 (39.8)	6.7 (41.9)
	4.2	1.1 (5.5)						2.4 (7.0)	
V	5.1	0.9 (5.9)		1.4 (10.9)	1.1 (11.5)				
	5.2						1.3 (5.6)		2.2 (4.8)
	5.3	1.9 (21.0)		1.9 (18.2)	1.0 (8.6)	1.4 (12.9)	1.7 (9.7)		
VI	6.1								−2.0 (2.5)
	6.2							−2.2 (6.8)	
VII	7.1	1.7 (10.1)	2.7 (13.4)	2.1 (14.1)	1.3 (8.2)	1.0 (6.7)	2.6 (12.2)	5.3 (22.5)	4.5 (9.7)
VIII	8.1	1.3 (13.1)	2.1 (17.5)		1.2 (15.4)	0.8 (6.4)	1.3 (6.1)		
IX	9.1								1.6 (2.5)
	9.2						1.0 (3.9)		
Total		10.3 (83.1)	6.5 (41.6)	7.9 (61.2)	6.4 (61.4)	4.2 (34.7)	11.6 (64.4)	17.1 (88.1)	19.1 (65.1)

Values in parentheses are percentage variation explained for individual QTL from each trial and for the totals of each trial.

The additive effect on a per QTL basis across individual trials ranged between 0.6 days (QTL 3.1 in F2-OK) and 6.7 days (QTL 4.1 in the GC-OK trial; Table 3). The total additive effects on a per trial basis were 6.5−10.3 days in the greenhouse trials, 4.2−11.6 days in the field trials, and 17.1-19.1 days in the growth chamber trials. Total additive effects ranged from 29 to 188.8% of the difference in days to heading between the parents (Table S2).

The eight trials were grown under varying conditions in the greenhouse, field, and growth chamber. QTL7.1 was found in seven trials, QTL2.2, 4.1, and 8.1 in five trials, QTL5.3 in four trials, QTL5.1 in three trials, and QTL3.1, 4.2, and 5.2 in two trials. Four genomic regions with QTL identified from multiple trials (QTL3.1, 5.1, 5.3, and 8.1) lacked significant QTL from the two trials carried out under the shortest photoperiod in the growth chambers, but variation due to different photoperiods and other environmental effects in the trials may be confounded.

For most QTL, the domesticated *S. italica* alleles increased days to flowering, as expected given that *S. italica* flowered much later than *S. viridis*. However, for three of the 16 total flowering time QTL identified (QTL 2.1, 6.1, 6.2), *S. viridis* increased days to flowering, although each of these was only identified in a single trial.

### Epistasis

A total of four significant epistatic interactions at the *P* < 2.3 × 10^−7^ level were detected in five of the eight trials conducted (Table 4). Two epistatic interactions were consistently identified in two trials and the other two were identified in a single trial. Epistatic interactions were detected between markers located in and outside of QTL regions as well as within and between linkage groups, and explained between 11.4 and 13.4% of the variance.

**Table 4:** Significant epistatic interactions between markers in the Setaria genome, and whether these markers colocalize with QTL regions

Marker 1	Linkage Group and Position in cM	QTL Present?	Marker 2	Linkage Group and Position in cM	QTL Present?	Interaction Was Observed in	% Variation Explained
UGSF827	III-42.4	No	UGSF867	III-65.1	No	F1-OK, F2-OK	11.4/13.2
UGSF436	I-50.1	QTL1.1	UGSF578	VIII-64.0	No	F2-GA, GC-BT	11.7/11.7
b112	I-51.4	QTL1.1	UGSF579	VIII-65.2	No	F2-GA	13.4
c562	IV-40.3	QTL4.1	b185-1	VII-110.6	No	GC-OK	11.8

### Comparison of QTL regions

**Figure 3:**
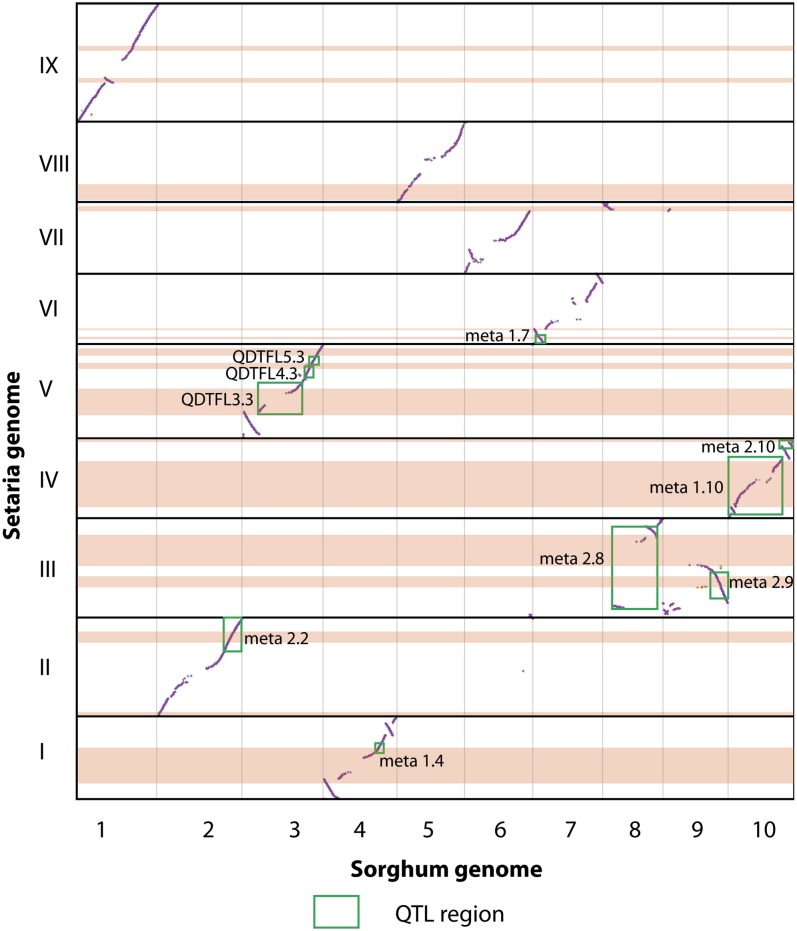
Whole genome dotplot of Setaria *vs.* sorghum. Diagonal purple lines in each cell indicate regions of synteny between the two genomes. Horizontal pink bars indicate genomic extent of Setaria QTL, whereas green boxes indicate QTL and meta-QTL for flowering time from sorghum (Mace and Jordan 2011) that are syntenic with Setaria QTL. Labels for QTL identified from sorghum are the same as in the original paper (Mace and Jordan 2011).

**Figure 4:**
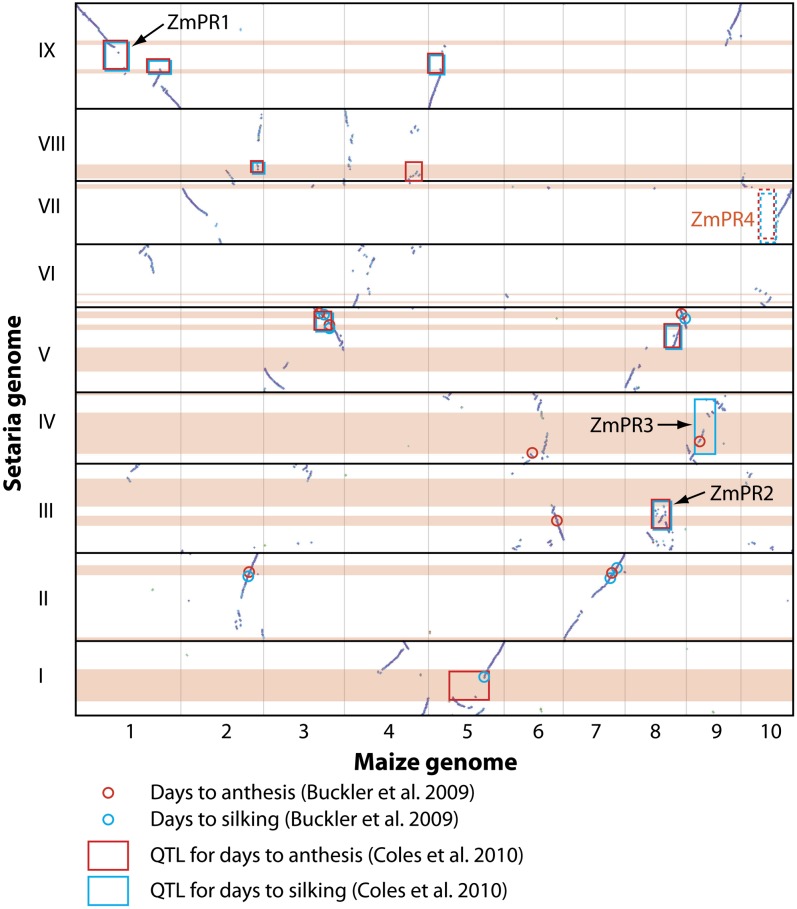
Whole-genome dotplot of Setaria *vs.* maize. Diagonal purple lines in each cell indicate regions of synteny between the two genomes. Horizontal pink bars indicate genomic extent of Setaria QTL, while boxes (Coles *et al.* 2010) and circles (Buckler *et al.* 2009) indicate QTL from maize that are syntenic with Setaria QTL. Red boxes and circles indicate days to anthesis, blue boxes and circles indicate days to silking. ZmPR1, ZmPR2, ZmPR3, ZmPR4 represent the four most important QTL regions in maize meta-analyses (see text). ZmPR4 (in orange) is represented by a box made of dashed lines as it is the only one that is not syntenic with a Setaria QTL.

To compare QTL among the three species we used a meta-analysis of sorghum QTL trials (Mace and Jordan 2011), a joint multiple population analysis of photoperiod sensitivity in maize (Coles *et al.* 2010) and the NAM maize QTL study (Buckler *et al.* 2009). All three of these studies used SNP markers that could be unambiguously placed on the physical genome sequence, and in all three studies some of the identified QTL were syntenic with the QTL found in Setaria. Comparison of the syntenic dotplots shows that there is less genome rearrangement between sorghum and Setaria than between Setaria and maize (Figures 3 and 4).

**Figure 5:**
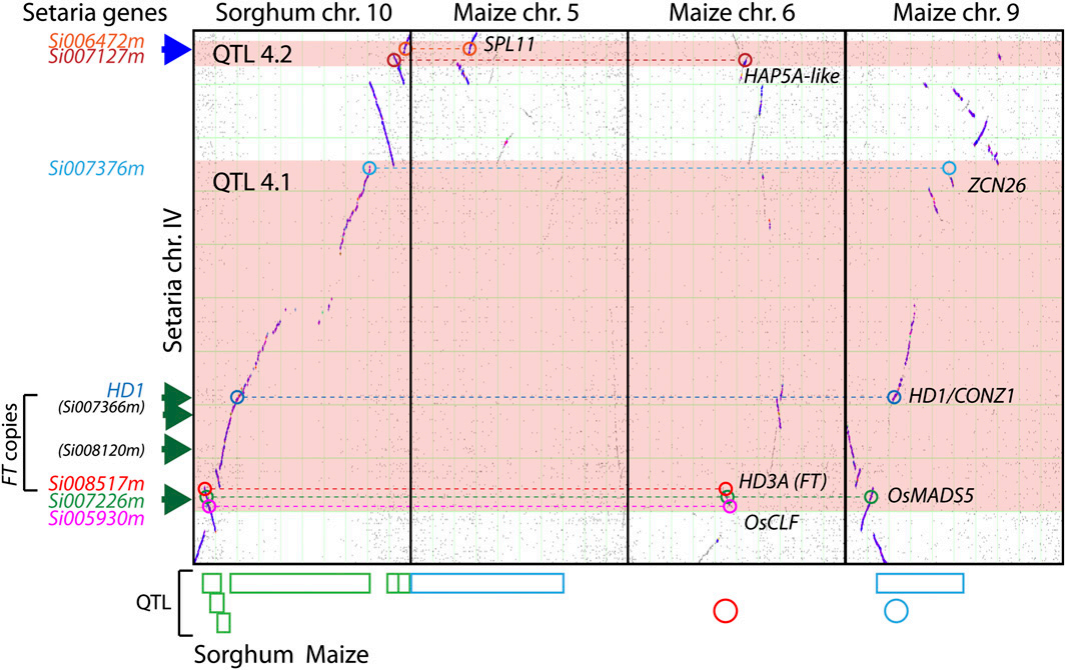
Analysis of Setaria chromosome IV and corresponding syntenic regions in sorghum and maize. Each panel represents a chromosome by chromosome dot-plot of Setaria chr. IV (vertical axis) *vs.*, in turn, sorghum chr. 10, maize chr. 5, maize chr. 6, and maize chr. 9 (horizontal axis). Horizontal pink bars indicate genomic extent of Setaria QTL4.1 and 4.2. On the bottom axis are QTL regions identified in sorghum (Mace and Jordan 2011) and maize (Buckler *et al.* 2009; Coles *et al.* 2010). Sorghum QTL are in green boxes, maize QTL are in red or blue boxes (Coles *et al.* 2010) or circles (Buckler *et al.* 2009), with red indicating days to anthesis and blue indicating days to silking. Syntenic candidate genes identified in the Setaria sequence (*SPL11*, *HAP5A-like*, *ZCN26*, *HD1*, *HD3A*, *OsMADS5*) were mapped onto each of the syntenic regions in sorghum and maize using COGE. On the left of the graph are the Setaria proteins identified as orthologous to the candidate genes from other species. Three of the Setaria proteins are co-orthologs of HD3A/RFT1 (FT) in rice, but only Si008517 is in synteny with the four chromosomes from sorghum and maize. The other two co-orthologs, Si07366m and Si08120m, are not found in syntenic regions. HD1 is not annotated in the Setaria genome, thus there is no numbered Setaria protein associated with it. Four dark green arrows on the left hand side of the graph indicate the approximate position of the four LOD peaks in QTL 4.1 and one blue arrow the single LOD peak in QTL4.2.

Genomic rearrangements were investigated in detail between Setaria chromosome IV and the corresponding sorghum and maize chromosomes (Figure 5). Four major syntenic blocks could be identified for the common region of QTL 4.1, three of which contain orthologous candidate genes. The genes have maintained, for the most part, their syntenic relationships, although there is evidence of partitioning of genes between the different maize chromosomes. QTL4.1 contains four LOD peaks, with two of these aligned with candidate genes (Figure 5). QTL4.2 is smaller, and has only a single LOD peak. Other QTL that show multiple LOD peaks include QTL2.2 and 8.1.

### Candidate genes

Several of the QTL regions contain candidate flowering pathway genes identified from rice, maize, sorghum, and Arabidopsis (Table 5). Identified candidate genes come from both autonomous and photoperiod-sensitive pathways, and span processes from initial light sensing (*CRY2*), coordination/regulation by the circadian clock (*PRR95*, *PRR59*, *GI*), the *CONSTANS* photoperiod pathway, the integration of flowering pathways (*FT*/*HD3A*/*RFT1*), and control of the transition of the shoot apical meristem from vegetative to flowering (*FD*/*Dlf1*, *TFL1*/*RCN1*). Phase change genes such as *SQUAMOSA PROMOTER BINDING PROTEIN-LIKE 9* (*SPL9*) and *SQUAMOSA PROMOTER BINDING PROTEIN-LIKE 11* (*SPL11*), and the microRNA *miR156* were also identified as candidates for differences in flowering time. Some QTL regions identified in only a single environment lack obvious candidate genes involved in control of flowering time (*i.e.*, QTL 2.1, 6.1, 6.2 and 9.1; Table 5). Interestingly, no QTL colocalize with genes involved in the vernalization pathway described from pooid grasses, suggesting that Setaria does not employ vernalization to cue flowering time.

**Table 5:** QTL number, interval (cM) for the region (entire interval for single QTL, overlapping common region for multiple QTL), flanking markers, marker at maximum LOD peak, and candidate genes within each QTL interval

QTL	Interval, cM	Flanking Markers	Marker at LOD Peak	Candidate Genes Colocalizing With Setaria QTL	Reference
1.1	48.1−60.1	MPGA8- UGSF467	UGSF436	*OsARP6/ZmARP6 (Si017263m)*	(Deal *et al.* 2005)
				*OsRCN2/ZCN2 (Si020516m)*	(Nakagawa *et al.* 2002; Danilevskaya *et al.* 2008)
				*ZmFPF1-like (Si018761m)*	(Kania *et al.* 1997; Melzer *et al.* 1999; Smykal *et al.* 2004)
				*LWD1(Si019593m)*	(Wu *et al.* 2008)
2.1	0.01−1.29	UGSF158- UGSF160	UGSF160	No candidate genes identified	
2.2	65.9−75.1	UGSF242- UGSF249	UGSF248	*SPL9-like (Si030195m)*	(Schwarz *et al.* 2008; Wang *et al.* 2009)
				*OsFTL4/ZCN18 (37,127,350-37,132,487)*	(Danilevskaya *et al.* 2008)
				*OsFPA (Si028909m)*	(Schomburg *et al.* 2001; Baurle *et al.* 2007)
				*OsPRR95 (Si029202m)*	(Murakami *et al.* 2003, 2005)
				*FD/DLF1 (Si031077m)*	(Neuffer *et al.* 1997; Muszynski *et al.* 2006)
				*OsMADS8/ZmMADS27 (Si030960m)*	(Kang *et al.* 1997)
3.1	66.9−78.2	UGSF864-UGSF848	UGSF850	*HAP3B-like (Si023400m)*	(Cai *et al.* 2007; Chen *et al.* 2007)
3.2	84.2−91.4	UGSF837-UGSF800	UGSF799	*REF6-like (Si024583m)*	(Noh *et al.* 2004)
4.1	42−53.1	UGSF896-UGSF961	UGSF914	*OsCLF (Si005930m)*	(Luo *et al.* 2009)
				*OsMADS5 (Si007226m)*	(Kang and An 1997; Jeon *et al.* 2000)
				*OsMADS55 (Si007357m)*	(Lee *et al.* 2012)
				*Hd3a(OsFTL2)/OsRFT1(OsFTL3)/ZCN15 (Si007366m/Si008517m/Si008120)*	(Kojima *et al.* 2002; Tamaki *et al.* 2007; Komiya *et al.* 2008)
				*HD1/CONSTANS (12,555,166-12,557,473)*	
				*CONSTANS-like (Si006432m)*	(Putterill *et al.* 1995; Yano *et al.* 2000)
				*OsCRY2 (Si006039m)*	(Hirose *et al.* 2006)
				*OsFTL12/ZCN26 (Si007376m)*	(Danilevskaya *et al.* 2008)
4.2	78.5−82.3	UGSF987- UGSF988	UGSF988	*HAP5-like (Si007127m)*	(Ben-Naim *et al.* 2006; Wenkel *et al.* 2006)
				*Zm-SPL11 (Si006472m)*	(Xie *et al.* 2006; Li *et al.* 2012)
5.1	44.7−65.1	UGSF300- UGSF335	UGSF289	*OsGI (Si000107m)*	(Hayama *et al.* 2003)
				*miR156F (cg1) (11,531,587-11,532,171)*	(Wu and Poethig 2006; Chuck *et al.* 2007)
				*OsFTL1/ZCN14 (13,585,697-13,586,064)*	(Izawa *et al.* 2002; Danilevskaya *et al.* 2008)
				*Hd17/OsELF3 (Si000443m)*	(Matsubara *et al.* 2008, 2012)
5.2	82.3−92.4	UGSF358- UGSF365	UGSF364	*FVE/OsFVE (Si001403m)*	(Ausin *et al.* 2004; Baek *et al.* 2008)
				*OsLFL1 (Si004459m)*	(Peng *et al.* 2007, 2008)
				*OsSPA1 (Si000378m)*	(Laubinger *et al.* 2006)
				*OsFTL9/ZCN12 (Si005012m).*	(Danilevskaya *et al.* 2008)
5.3	98.3−113.8	UGSF379-UGSF388	UGSF387	*REF6 (Si000062m).*	(Noh *et al.* 2004)
6.1	35.2	UGSF690	UGSF690	No candidate genes identified	
6.2	39.7−43.4	UGSF686-p10	p10	No candidate genes identified	
7.1	83.1−101	UGSF665-UGSF778	b200	*APETALA2 (Si012635m)*	(Bowman *et al.* 1989; Kunst *et al.* 1989)
				*HAP5-like (Si012136m)*	(Ben-Naim *et al.* 2006; Wenkel *et al.* 2006)
8.1	24.5−41.4	b185_2- UGSF526	UGSF513, UGSF525	*OsTFL1/OsRCN1/ZCN1/TFL1 (Si028168m)*	(Shannon and Meekswagner 1991; Gustafsonbrown *et al.* 1994; Nakagawa *et al.* 2002)
				*OsPRR59(Si026170m)*	(Murakami *et al.* 2003, 2005)
9.1	80.4−81.0	UGSF77- UGSF79	UGSF77	No candidate genes identified	
9.2	83.4−86.4	UGSF93- p91	p91	*REF6-like (Si034241m)*	(Noh *et al.* 2004)

## Discussion

### Phenotypic variation

The eight trials varied in a number of environmental variables, including photoperiod, temperature, light intensity, planting density, and water availability. Several of these, such as photoperiod and temperature, are known to have effects on flowering time (Garner and Allard 1920; Robertson 1968; Koornneef *et al.* 1991; Chew *et al.* 2012; Kim *et al.* 2012). In fact, early work on *S. viridis* found that earliest flowering was under a photoperiod of 8 hr light, and that a lower temperature of 22.5° led to faster flowering than at 30° (Schreiber and Oliver 1971). We too observed variation across the eight trials in flowering time of the parents (although *S. viridis* always flowered earlier than *S. italica*), and in the order in which the RILs flowered, but it is hard to assign these differences to the effect of any one environmental variable. Overall, increasing the day length delayed flowering of both the RILs and the parents of the cross. Furthermore, the growth chamber trial of the parents at 12 and 16 hr light demonstrated that both parents flowered earlier under a 12-hr regime. However, factors other than day length were likely responsible for the earlier heading of *S. italica* in the GA compared with the OK field trials, and for the delay in flowering observed in the OK compared with the BT growth chamber trial, as those trials were grown under similar photoperiod regimes. Much work needs to be done in order to understand how combinations of different environmental variables affect flowering time in these genotypes.

### QTL analyses

Despite differences in management, experimental design, and environmental conditions of the eight trials, nine out 16 QTL were identified in at least two trials. In most cases the *S. italica* allele acted to increase days to flowering, as might be expected from the later flowering time of *S. italica* in all trials. However, for three of the 16 flowering time QTL, *S. viridis* alleles increased days to flowering. Very few epistatic interactions were detected, although our stringent Bonferroni-corrected false-discovery rate may have precluded real but less significant interactions. Of the four interactions discovered, one was between two markers outside of any QTL region, and three were between markers where one was in a QTL region and the other was not. No interaction was identified between two markers where both markers were in QTL regions (Table 4). In addition, epistasis was not seen in all trials, suggesting that such interactions are not a prominent feature of this data set. This is in agreement with earlier experiments on the F3 generation of this cross, which uncovered a similar pattern of interaction between the RFLP markers used to construct the map (Doust *et al.* 2004, 2005; Doust and Kellogg 2006).

In general, the analysis of syntenic blocks between Setaria and maize and sorghum reveals a more-or-less 1:1 relationship between Setaria and sorghum chromosomes but a more complicated set of relationships between Setaria and maize. This is no doubt due to the fractionation of the maize genome following the whole genome duplication 9−12 mya and its subsequent diploidization (Blanc and Wolfe 2004; Swigonova *et al.* 2004). Therefore, a single QTL region in Setaria may be dissected into syntenic blocks that occur on two maize chromosomes. In both of the comparisons, we found that single Setaria QTL will usually be comprised of multiple syntenic blocks when compared with either sorghum or maize. QTL4.1 (Figure 5) is a case in point, where at least two inversions have occurred in Setaria compared to the corresponding region on sorghum chromosome 10 (Figure 5, this article; Figure 11 in Bennetzen *et al.* 2012). The candidate genes that colocalize to this QTL region maintain local order on each syntenic block, but genomic inversions have resulted in an overall rearrangement compared to Setaria. The partitioning of syntenic regions between different chromosomes in maize has resulted in further dissociation of these genes, with most genes confined to one or other homeologous chromosome, although others such as OsMADS5 have copies on both maize homeologs.

The presence of multiple syntenic blocks within a single Setaria QTL region is correlated with the presence of multiple LOD peaks in a number of the QTL intervals (Figure 5, Figure S2), implying that these QTL cover regions where multiple genes may be involved in flowering time regulation. Candidate genes in these intervals are often colocated with one or other of these peaks, as can be seen for QTL4.1, a large QTL interval on Setaria chromosome IV (Figure 5).

Meta-analysis of QTL studies in maize has revealed a number of genomic regions where QTL for multiple traits are colocalized. Studies have recognized from four to six regions, on chromosomes 1, 8, 9, and 10 (Chardon *et al.* 2004; Coles *et al.* 2010; Xu *et al.* 2012). Four of these regions were shown to account for a high proportion of the variance in flowering time, with three (labeled ZmPR1, ZmPR2, and ZmPR3) accounting for approximately 10% each and the last (ZmPR4) accounting for approximately 40% (Coles *et al.* 2010). The genomic comparison of QTL in Setaria and maize reveals that ZmPR1, ZmPR2, and ZmPR3, on maize chromosomes 1, 8, and 9, are syntenic to QTL regions in Setaria, but that ZmPR4, the largest effect QTL region in maize on chromosome 10 (Coles *et al.* 2010), is not. However, there were also Setaria QTL without syntenic QTL in the other two species, suggesting that control of flowering time may involve both conserved and novel genetic loci in any one species. Such a pattern has been demonstrated for other traits such as shattering, where one gene, SHATTERING 1, appears to underlie QTL in multiple species (Lin *et al.* 2012), whereas most other genes are confined to individual species (Li and Gill 2006). Conservation of flowering time QTL across grasses for at least some loci was noted first several years ago, and our study demonstrates support for those earlier findings (Paterson *et al.* 1995).

### Candidate genes

It is somewhat surprising that the high level of conservation in QTL position between Setaria and sorghum and maize is not reflected by conservation of the cloned flowering time genes from sorghum and maize as candidates for those QTL. Only three genes, DLF1 (FD), GI, and CONZ1 (CO), were identified in Setaria QTL intervals. Major effect genes such as ZmCCT, VGT1, and PRR37 did not colocalize with Setaria QTL. This lack of correspondence may either indicate real differences between the three species, or may simply reflect the relatively poor knowledge of genes underlying flowering variation in panicoid grasses. The latter seems likely because of the substantial number of candidate genes from rice and Arabidopsis that colocalize in the QTL intervals. In addition, most emphasis in flowering time studies in maize and sorghum has been on the effect of photoperiod, whereas the Setaria trials described here only sample a small range of photoperiods. The conservation of candidate genes across the three species in QTL such as QTL 4.1 likely indicates that genetic control of flowering is similar between the three species.

In contrast to maize, sorghum, and rice, Setaria appears to have been domesticated away from the tropics, in the northern temperate region of China (Bettinger *et al.* 2010). Furthermore, annual Setaria species in these regions germinate and grow in the spring and summer, and do not appear to need vernalization to successfully flower. This is supported by our failure to find genes such as VRN1 and VRN2 from the grass vernalization pathway colocalized with Setaria QTL. In contrast, a number of candidate genes were from the CONSTANS photoperiod pathway (SPA1, CO/HD1/CONZ1, HAP3-like, HAP5-like), as well as genes proposed to be involved in autonomous or endogenous pathways (FVE, FPA). Surprisingly, only two candidate genes (LFL1 and OsMADS5) from the alternative photoperiod pathway in rice (centered on EHD1) colocalized with Setaria QTL, perhaps due to the limited range of photoperiods sampled. The presence of this pathway in maize has already been established with the discovery of a GHD7 ortholog (ZmCCT) (Hung *et al.* 2012), and we predict that this pathway will prove to be present in Setaria as well.

A number of circadian clock elements colocalized to Setaria QTL, including *PRR59*, *PRR95*, and *GI*, as well as genes that closely interact with these, such as *ELF3-like* and *ELF4-like* genes (Higgins *et al.* 2010; Yang *et al.* 2012). *GI* has multiple roles, including directly interacting with *miR172* to control phase change independent of photoperiod (Jung *et al.* 2007) and with *CONSTANS* (*CO*) in the main photoperiodic flowering time pathway (Hayama *et al.* 2003). The two pseudoresponse regulators PRR59 and PRR95 are related to PRR37, which underlies the major flowering time locus *Ma1* in sorghum (Murphy *et al.* 2011), but PRR37 was not located in any of the identified QTL. It is possible that modifications to any of these circadian clock elements can be used to produce differences in flowering time. Interestingly, the PRR37 ortholog in maize does colocalize to a QTL for flowering time, potentially creating a difference in control of flowering time between Setaria and the other two species. Studies in switchgrass (*Panicum virgatum*), a closely related species to Setaria, may reveal whether these differences are diagnostic for the different tribes to which maize and sorghum (Andropogoneae) and Setaria and switchgrass (Paniceae) belong.

Setaria has three copies of *FT* in QTL4.1 that are co-orthologous to HD3A and RFT1 in rice, but only HD3A and one of the Setaria copies (Si08517m) are in synteny. FT is part of the large PEBP protein family, which has undergone several rounds of duplication in grasses as compared to Arabidopsis (Chardon and Damerval 2005), and Setaria has 22 PEBP proteins (Bennetzen *et al.* 2012). In maize, ZCN8 has been described as the functional FT homolog, identified through expression analysis (Danilevskaya *et al.* 2008), but the Setaria ortholog of ZCN8 did not colocalize to any of the QTL intervals. Another subclade of the PEPB protein subfamily contains TERMINAL FLOWER-like (TFL) genes, which act antagonistically to *FT* in Arabidopsis to prolong the vegetative phase of the apical meristem (Kardailsky *et al.* 1999; Kobayashi *et al.* 1999). A TFL1 homolog was found in Setaria QTL8.1.

Lastly, a number of genes involved in phase change and the regulation of the length of the vegetative growth phase were also identified (*SPL9*, *SPL11*, *miR156*), suggesting that phase change and flowering time are intimately connected. Recent work on flowering time regulators such as GI and APETALA1 have uncovered an intricate coupling of phase change and flowering time pathways (Yant *et al.* 2010; Huijser and Schmid 2011), indicating that a more holistic view of gene regulation is necessary to understand flowering time in plants.

The eight trials in this study showed remarkable similarities in the position of QTL for flowering, given the diversity of the growing conditions. This finding suggests that the integration of flowering pathways performed by the plants provides considerable buffering against environmental change. However, the presence of a number of QTL regions found only in the longer-day environments suggests that, in common with other grasses, long days likely suppress flowering. This was corroborated by the later flowering time of the parents under 16-hr light than under 12-hr light, when both were grown in a controlled environment. The candidate genes identified are mainly from the *CONSTANS* photoperiod pathway but also include members of the *EHD1* photoperiod and autonomous pathways. We did not explore the influence of phase change on flowering time, but the presence of several candidate genes for phase change suggests that this will be a fruitful area of investigation. The presence of genes conserved across grasses and dicots in the syntenic QTL regions between Setaria, maize, and sorghum suggests that an ancestral flowering time pathway involving CONSTANS is conserved across grasses, while the absence of many of the cloned genes of major effect from maize in these same QTL suggests that each species has undergone further lineage specific evolution in genetic control of flowering time. We have identified several genome regions based on the QTL analyses and have embarked on fine-mapping loci using a combination of hybrid inbred families and gene expression surveys.

## Supplementary Material

Supporting Information
