# Supplementary material for: Genetic Control and Comparative Genomic Analysis of Flowering Time in Setaria (Poaceae)
Source: G3 (Bethesda). 2013 Feb 1;3(2):283–95. doi: 10.1534/g3.112.005207 (PMC3564988; doi:10.1534/g3.112.005207)
Supplement: Supporting Information [file supp_3_2_283__index.html]

Supporting Information 

# Genetic Control and Comparative Genomic Analysis of Flowering Time in Setaria (Poaceae)

## Supporting Information for Mauro-Herrera *et al.*, 2013

**Files in this Data Supplement:**

- Supporting Information - Figures S1 and S2, Tables S1 and S2, and Files S1 and S2 (PDF, 886 KB)
- Figure S1 - Full QTL map with all QTL found (PDF, 436 KB)
- Figure S2 - LOD graphs for each of the eight trials (PDF, 322 KB)
- Table S1 - Primers sequences, detection method, and Genbank accession number for STS markers (PDF, 175 KB)
- Table S2 - Differences between the parents of the cross in individual trials (PDF, 78 KB)
- File S1 - Supporting Information (PDF, 175 KB)
- File S2 - Genotype and phenotype information for the genetic map and QTL analyses (.csv, 263 KB)
