# Supplementary material for: Genetic Control and Comparative Genomic Analysis of Flowering Time in Setaria (Poaceae)
Source: G3 (Bethesda). 2013 Feb 1;3(2):283–95. doi: 10.1534/g3.112.005207 (PMC3564988; doi:10.1534/g3.112.005207)
Supplement: Supporting Information [file supp_3.2.283_005207SI.pdf]

G3 · Genes | Genomes | Genetics

GENETIC CONTROL AND COMPARATIVE GENOMIC ANALYSIS OF FLOWERING TIME IN SETARIA (POACEAE)

Margarita Mauro-Herrera[*], Xuewen Wang[§,**], Hugues Barbier[§§], Thomas P. Brutnell[§§,***], Katrien M. Devos[§], Andrew N. Doust[*]

Affiliations:
* Department of Botany, Oklahoma State University, Stillwater, OK 74078
§ Department of Crop and Soil Sciences, and Department of Plant Biology, University of Georgia, Athens, GA 30602
** China Tobacco Gene Research Center, No.2 Fengyang Street, High-Tech Zone, Zhengzhou, P. R. China Postcode: 450001
§§ Boyce Thompson Research Institute, Cornell, Ithaca, NY 14853
*** Donald Danforth Plant Science Center, St. Louis, MO 63132

DOI: 10.1534/g3.112.005207

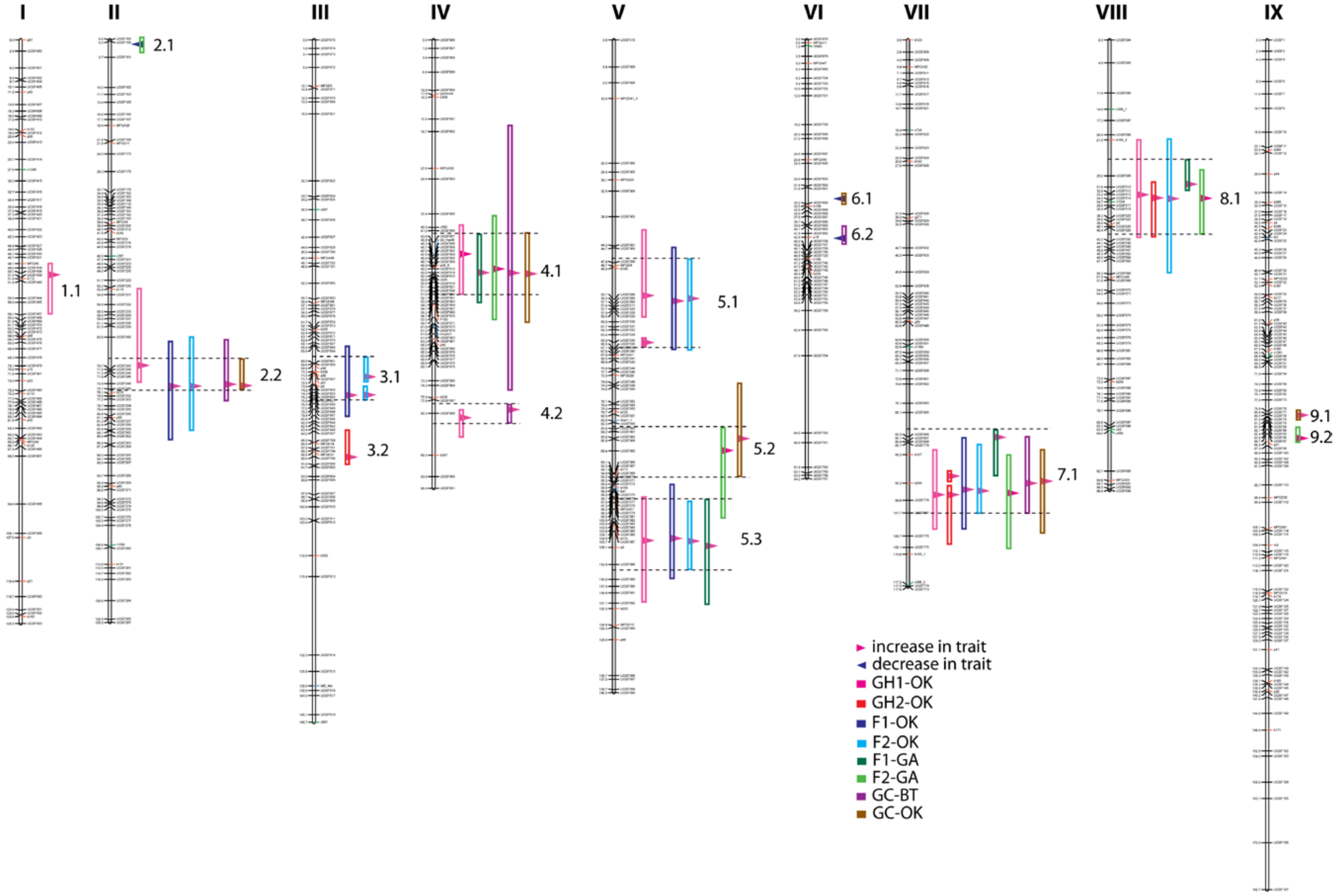

Figure S1 Full QTL map with all QTL found. Dashed black lines delimit common QTL regions defined by overlapping QTL intervals. Candidate genes were examined for common regions for QTL identified in multiple trials and for the entire interval for QTL found in a single trial. GH = Greenhouse, GC = Growth Chamber, F = Field. OK = Oklahoma State University, Stillwater, OK; BT = Boyce Thompson Institute, Ithaca, New York; GA = University of Georgia, Athens, GA.

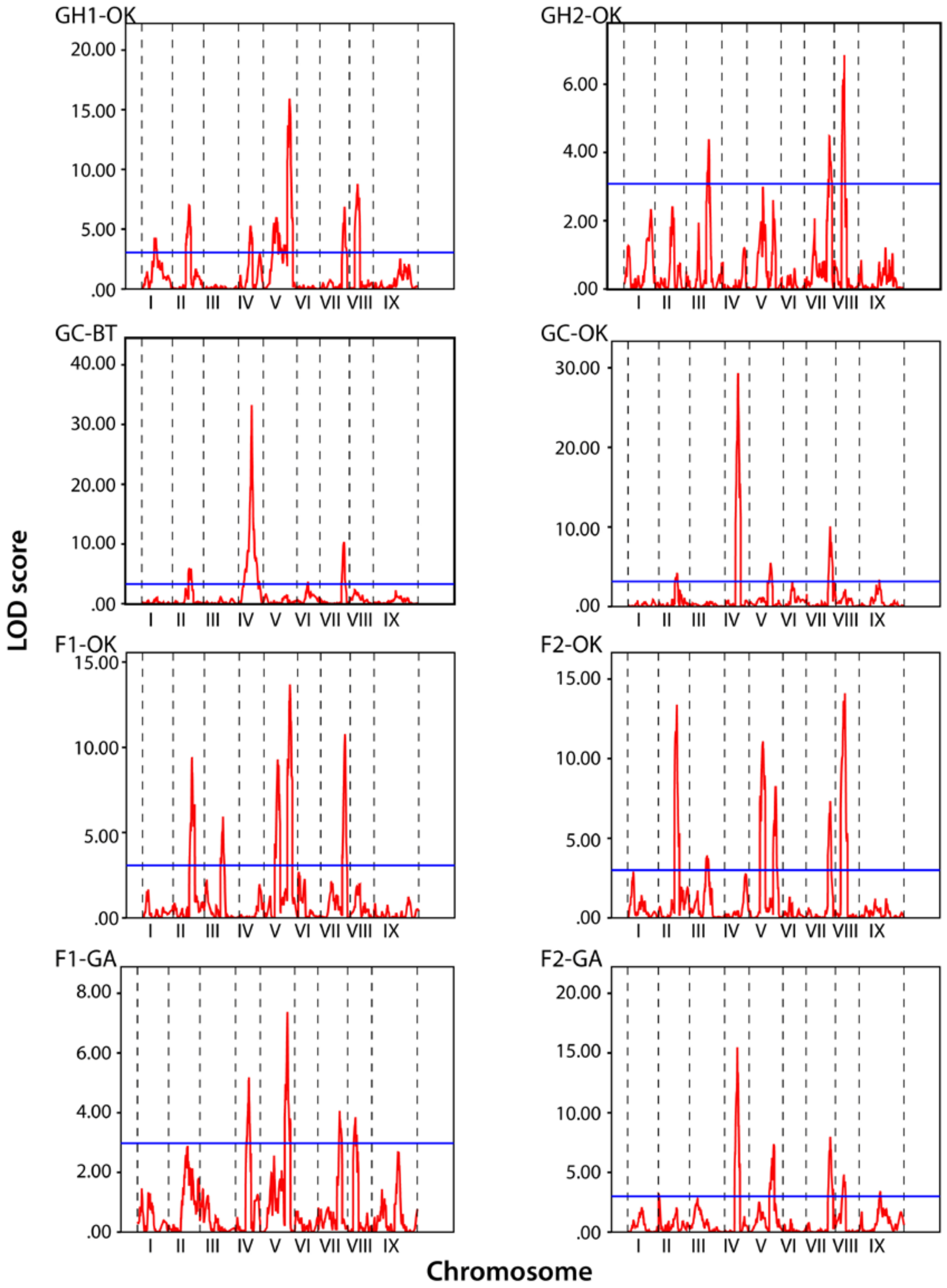

Figure S2 LOD graphs for each of the eight trials. Each graph shows the LOD scores for each trial along each chromosome in Setaria, with chromosomes lined up end to end (I-IX). The horizontal blue line represents the $P < 0.05$ significance level, so that QTL are declared significant if the peaks are above that line. In several cases there are multiple peaks underlying a single QTL. GH = Greenhouse, GC = Growth Chamber, F = Field. OK = Oklahoma State University, Stillwater, OK; BT = Boyce Thompson Institute, Ithaca, New York; GA = University of Georgia, Athens, GA.

File S1

Supporting Information

1. Plant materials, experimental design and growing conditions

Greenhouse Trials: The first greenhouse trial (GH1-OK) was grown in 2008 in a greenhouse without additional lighting at Oklahoma State University during the months of June to September. Day length varied between 13.8 and 14.3 hours, with an average daily temperature of 26 °C, and light intensity of 1400 μmol $cm^{-2}s^{-1}$. The experimental design consisted of a Randomized Complete Block Design (RCBD) with six blocks and one replicate of each RIL per block. RILs were planted in 11x11x11 cm square pots (approximately 1210 cm^3), filled with Metro-Mix 366 (Sun Gro Horticulture Canada Ltd.) and spaced 15.5 cm apart. Plants were irrigated as needed with an aqueous complete fertilizer mix (Jack's mix: Nitrogen, Phosphorous and Potassium - 20:20:20).

The second greenhouse trial (GH2-OK) was grown in 2010 with additional metal halide and high-pressure sodium lighting at Oklahoma State University during the months of July to September. Day length, consisting of natural light with supplemental lighting before dawn and after dusk, was 16 hours, with an average daily temperature of 26.5 °C, and light intensity of 1400 μmol $cm^{-2}s^{-1}$. In this trial only 107 RILs were evaluated due to limitations in space. The experimental design consisted of a RCBD with four blocks, two planted at high density (11 cm apart) and two at low density (31 cm apart), with one replicate of each RIL per block. RILs were planted in 11x11x14 cm square pots (approximately 1573 cm^3), filled with Metro-Mix 366 (Sun Gro Horticulture Canada Ltd.). Plants were irrigated as needed with an aqueous complete fertilizer mix (Jack's mix: Nitrogen, Phosphorous and Potassium (20-20-20)).

Field Trials: Two trials were conducted at the Cimarron Valley Field Research Station in Perkins, Oklahoma during the months of May to July of 2010 and 2011. Seed was germinated in a greenhouse and transplanted to the field two weeks after sowing, at approximately the two-leaf stage. Day length varied between 14 and 14.3 hours, with an average daily temperature of 26.5˚C in 2010 and 28˚C in 2011. The first of these Oklahoma field trials (F1-OK) consisted of a RCBD with two blocks, with three replicates of each RIL per block. RILs were planted 25 cm apart from each other in rows. In order to mitigate the variable competition effects stemming from RILs of different sizes growing together, RIL rows were separated by rows of border plants, with rows 25cm apart. A commercial foxtail millet variety (German millet) was used for the borders and plants were watered as needed. One nitrogenous fertilizer application (as urea) was done in the sixth week of growth at a rate of 50 lb/acre (56 kg/ha). The second trial (F2-OK) was also a RCBD with four blocks, two planted at high density (15 cm apart) and two at low density (60 cm apart), with four replicates of each RIL per block. German millet was used as border plants, as in the first field trial. Blocks were pretreated with nitrogenous fertilizer (as urea) at a rate of 50 lb/acre (56 kg/ha), and with pre-emergent herbicide (Prowl® H2O herbicide, BASF Corp.).

Two field trials were also conducted at the University of Georgia, Athens, in June-July 2011 and July-August 2011 (14.3 hours day length, average temperature of 28 ^{0}C). These trials are labeled F1-GA and F2-GA. For the first trial (F1-GA), seeds were sown in soil filled peat pots (1 seed/pot). The pots were placed at 4 ^{0}C for 1 week after which they were placed in the glasshouse under natural light conditions. Twenty days later, the plants were planted in the field in hill plots in a RCBD with two blocks. For the second trial, ~100 seeds per RIL were sown in soil in pots, placed at 4 ^{0}C for 4 days and then in the glasshouse under 14.2 hours natural day light for four days. Two-day old seedlings were transplanted to soil-filled peat pots (1 plant/pot), and 10-day old seedlings were planted in hill plots in

a RCBD with two blocks. In both trials, hill plots were separated by 36” and each hill plot contained a single RIL in four replicates. Plots were irrigated when necessary.

Growth Chamber Trials: The first growth chamber experiment (GC-BT) was conducted at the Boyce Thompson Institute. Plants were grown under artificial light with 12 hours of light (750 μmol.m^{-2}.s^{-1}) and 12 hours of darkness. Average temperature was 31˚C during the light period and 25˚C during the dark. Five plants per RIL were planted in 220.5 cm^3 pots filled with metro mix 360 (Sun Gro Horticulture), and thinned to two plants 10 days after sowing. Fertilization was applied every 7 days during the experiment (N-P-K; 20-20-20).

The second growth chamber experiment (GC-OK) was conducted in 2012 at Oklahoma State University. Plants were grown under artificial light with 12 hours of light (350 μmol.m^{-2}.s^{-1}) and 12 hours of darkness. Average temperature was 28˚C during the light period and 22˚C during the dark. Only 126 RILs were evaluated in this experiment due to space limitations, with RILs chosen to represent the extremes of flowering time (data taken from previous greenhouse experiments). The experiment had a single block with three replications per RIL, with plants spaced 15 cm apart. RILs were planted in 11x11x14 cm square pots (approximately 1573 cm^3), filled with Metro-Mix 366 (Sun Gro Horticulture Canada Ltd.). Plants were irrigated as needed with an aqueous complete fertilizer mix (Jack’s mix: Nitrogen, Phosphorous and Potassium (20-20-20)).

Parental trial under different photoperiods: *Setaria italica* and *S. viridis* plants were grown in growth chambers at 28˚C day and 22˚C night temperatures. The long day photoperiod regime had 16 hours light while the short day regime had 12 hours light (350 μmol.m^{-2}.s^{-1}). Number of leaves on the culm, average speed of leaf initiation, time taken until inflorescence meristem starts to initiate, and time of inflorescence emergence were measured for each species in each environment. Plants were grown in 11x11x14 cm square pots (approximately 1573 cm^3), filled with Metro-Mix 366 (Sun Gro Horticulture Canada Ltd.), and irrigated as needed with an aqueous complete fertilizer mix (Jack’s mix: Nitrogen, Phosphorous and Potassium (20-20-20)).

2. Molecular Marker Development and Genotyping

Almost two hundred published SSR primer pairs (Jia *et al.* 2007; Jia *et al.* 2009; Gupta *et al.* 2012) were tested on the parents and a total of 126 informative markers were chosen to genotype the RIL population. PCR fragment separation for SSR markers was done via agarose gels (1 to 3 % depending on fragment sizes) or with an ABI PRISM 3730 Genetic Analyser (Applied Biosystems). Amplification reactions for SSR fragments separated in agarose gels were done in a 10 µl volume containing 0.5 Units of Taq DNA polymerase (Promega Corp), 1.5 mM $MgCl_2$, 100 µM dNTPs, 20 ng of genomic DNA, 2.0 µl of 5x GoTaq buffer (Promega Corp) and 0.32 µM of forward and reverse primer. Some primers required the addition of 5% DMSO (indicated DMSO in Supplementary Table1) to consistently amplify. PCR cycling conditions consisted of an initial denaturation step at 94 °C for 2 min followed by 35 amplification cycles with an initial touchdown, and a final 5 min extension cycle at 72 °C. Each of the 35 cycles had a denaturation step at 94 °C for 40 s followed by 40s at the annealing temperature which was started 4 °C above the primers’ melting temperature and decreased every two cycles by 2 °C to reach the primers’ annealing temperature by the fifth cycle, and an extension step of 1 min at 72 °C. PCR fragments were visualized in 1 to 3 % agarose gels stained with ethidium bromide. Amplification reactions for SSR markers analyzed with the ABI PRISM 3730 Genetic Analyser (Applied Biosystems) were done using the “economic method” developed by Schuelke (Schuelke 2000) and further improved by Missiaggia and Grattapaglia (Missiaggia and Grattapaglia 2006). PCR reactions were done essentially as described

above, except that primer concentrations were 0.10 µM for the forward tailed primer, 0.3 µM for the reverse primer and 0.2 µM for the fluorescently labeled forward primer

To develop sequence-tagged-site (STS) markers for selected rice RFLP probes/sequences used in the F_2 foxtail genetic map (WANG *et al.* 1998) and for genes of interest (Supplementary Table 1), we first designed minimally degenerate primers to consensus regions of the rice sequences and their homologs in maize, sorghum and sugarcane. Sequences were obtained from Gramene (http://www.gramene.org) and the NCBI website (NCBI http://www.ncbi.nlm.nih.gov/gene), and aligned using Sequencher version 4.9 (Gene Codes Corporation) or MacClade version 4.0 (Maddison and Maddison 2000). Primers were designed with the programs GeneFisher (http://bibiserv.techfak.uni-bielefeld.de/genefisher2/) (GIEGERICH *et al.* 1996) and Primaclade (http://primaclade.org) (GADBERRY *et al.* 2005), and tested for amplification in the parental genotypes *S. italica* accession B100 and *S. viridis* accession A10. Single band PCR products were TOPO TA cloned (Invitrogen CA), and sequencing was done following the standard protocol for BigDye Terminator v1.1 (Applied Biosystems). Sequence reads were analyzed with Sequencher 4.9 and SNPs or fragment length polymorphisms were identified between the two parental genotypes. These were detected using two primer pairs to amplify, respectively, the two different alleles of a SNP in the same PCR reaction, incorporating mismatches that increase specificity in the 3' end of the primers that end on the SNP polymorphism (tetraprime ARMS-PCR) (YE *et al.* 2001). Primers for the ARMS-PCR technique were designed with the program Primer1 available at http://cedar.genetics.soton.ac.uk/public_html/primer1.html (Supplementary Table 1). PCR reactions for the ARMS-PCR technique were done in a 10 µl final volume. Reagent concentrations were the same as those for the SSR amplifications, except for primer concentrations that were 0.84 µM for each of the two inner primers and 0.084 µM for each of the two outer primers. Some primer pairs required the addition of 5% DMSO (indicated DMSO in Supplementary Table1). PCR cycling conditions were the same as those used for SSR amplifications. PCR fragments were visualized in 2% agarose gels stained with ethidium bromide.

File S2

Genotype and phenotype information for the genetic map and QTL analyses

File S2 is available for download at http://www.g3journal.org/lookup/suppl/doi:10.1534/g3.112.005207/-/DC1.

Table S1 Primers sequences, detection method, and Genbank accession number for STS markers

Locus	Linkage group	Primer sequence (5′ →3′)	Ta °C	Fragment sizes (bp)	Polymorphism detection method	Genbank Accession Number
c1246	1	F inner: AAA GTG TCG TTT TAA TTA GTA CAG TTG TC	52	160-320	ARMs PCR	GF112102
*(Xrgc1246)**		R inner: TGA TTG CCA CAG AAC TAG TAT CTT TAT				GF112117
		F outer: GGA TAG ATG CTA TTC AGT AAG ACA CTA AA				
		R outer: TGC TTT TTC TTC AAC TAT GAT ATG TAT C				
c397	2	F inner: TTA TGT CTG CAA GTG ATC AAT TGT GAG TT	52	100-200	ARMs PCR	GF112103
*(Xrgc397)**		R inner: AAA TTC TGC ATG TAA AAT GAG TCC CG				GF112118
		F outer: GTG ATT CAC CTT CTC AGC TTA AGG CAT				
		R outer: GTA TTC CTG TGC AAT CTG TCT TAC TGC A				
r1789	2	F: TCG TTG GCG TGC CAC TGA AA	58	308	PCR amplicons	GF112104
*(Xrgr1789)**		R: TGG TCC TGC ACT CAG AAG CAC A			digested with	GF112119
					BsmF1 at 65 °C	
c901	3	F inner: ACT CCC ATT CTC TCG CGT TCC TTC G	64	80-140	ARMs PCR	GF112105
*(Xrgc901)**		R inner: CTG ACC CCT GGA GGC TGG GGT				GF112120
		F outer: AAG GAG GCT GGT GTC ACC TAT CAC CG				
		R outer: CCC TGT GCT AGA CAG GAT AGA CGG GGA				
BARREN	3	F: GGT ACG TGC AGG TTC TAC GC	56	254-257	3730 Fragment	GF112106
INFLORESCENCE 2 -		R: TTC AAC CGG AAC AGG TGG T			analysis	GF112121
LIKE (bif2-like)					DMSO	
c597	3	F inner: TAG CAG AGA TGA AAA GGT ATA GAA CAG CG	52	130-260	ARMs PCR	GF112107
*(Xrgc597)**		R inner: CTT TTC AAC GAC AAG ATT CAG GTA CGT				GF112122
		F outer: ATG CTT GAC AGG TAT ACC AGT GCA AG				

		R outer: TCT GTA TAA CAC TTG CAG GAA GGA CG				
c562	4	F inner: ACG CCA GGG ATT ATG TGA CTT TTT AG	60	130-250	ARMs PCR	GF112108
*(Xrgc562)**		R inner: TTC CTG CAA GAG TTT CAA TAT GAG GAT				GF112123
		F outer: CAG ATA GGA AGT AGC CAT TAA TGC CTG				
		R outer: AAG CTT GGT ATG AGA AAC GAG TGT GTC				
DWARF3 (D3)	4	F: GTC CCA CAG CGG GCA GTG TC	61	75-430	PCR amplicons	GF112109
		R: CGG CAT CCA CCA GCC AAG GG			digested with	GF112124
					HaeIII at 37 °C	
c235	4	F inner: GTT TCG ACT GCA AAA TTA TGA AAC TAC C	56	50-200	ARMs PCR	GF112110
*(Xrgc235)**		R inner: AGT TCT CAC TTC TCA AGT CCC AAA CA				GF112125
		F outer: GAA ATG AGA ACT ACC ATT CAA CAT TCG				
		R outer: AGA AAA ACC AAT TTC ATC AAA AGA ACG				
MONOCULM 1 (MOC1)	4	F inner: CCT CGA GCG CCT CGA ACA CCT CC	66	50-180	ARMs PCR	GF112111
		R inner: CCG TCG CCA TGG ACC ACT ACG CA			DMSO	GF112126
		F outer: AGC ACC TCC TGC TCC ACC GCC AG				
		R outer: GTG ACC GTC GCG GAG AGG GAG ATG AT				
BARREN STALK 1 (BA1)	5	F: CCA TCT TTC TTT GAT CCC TGT C	56	225-240	3730 Fragment	GF112112
		R: GGG CAG ATA TGG TCA TTT CAC			analysis	GF112127
MORE AXILLARY	5	F inner: GTC GGC TTT CTG GAC AGG CCG CAA	66	70-220	ARMs PCR	GF112113
BRANCHES 1 (MAX1)		R inner: CAG GCG GCA TTC GGC GCG TAC			DMSO	GF112128
		F outer: GTC ATC CCT GGC GTG GCT CTT CCC AT				
		R outer: CCA CCT TGC CGG ATT GAT TCC AAC CAT				
r1943	6	F inner: CAT CAT TCA CTG GTC CTA TAC ATG AG	56	150-300	ARMs PCR	GF112114
*(Xrgr1943)**		R inner: GAA ATG AGT CTG GAA GAA CAT GAA G				GF112129
		F outer: CGA ATA TAT AAC CCA CAA GAT GAA AAG				
		R outer: TAG CAA TAT TCA TTT TTA TTG GCA ATT				

c389_2	7	F: GAT CCC ATT GGT CTT GCA ACT	56	310-330	3% Agarose gel	GF112115
*(Xrgc389)**		R: ACA AGG TTC CAC CTC AAC CTG				GF112130
c1100	7	F: ATG AAT GCG GTG CTC TGT G	56	169-175	3% Agarose gel	GF112116
*(Xrgc1100)**		R: GGA AAA AGG GTG ACC TTC CT				GF112131
g271	7	F inner: GTC ATC GCC TAC AAC CCC ACA	59	75-190	ARMs PCR	GF112094
*(Xrgg271)**		R inner: TTG TCT TCT TCG GAC AGG GGG				GF112086
		F outer: GAC CTA CTG GTG GAA CAG CCA GAA				
		R outer: ACA AGA TGA GCA CGG AGT ATC ACT GAT				
c734	7	F inner: CTA TGC AAA GCT TTA TGT ATG CGA A	54	100-180	ARMs PCR	GF112095
*(Xrgc734)**		R inner: CCT CTA TAT ATG AAC AAC TTA ATT CTG				GF112087
		F outer: ATT TAG TCT GTA GTT ACA CCT ACT TCT GTG				
		R outer: ACA GAT ACC ATG AAG ATT AAA GAG TCA				
c950	8	F inner: TCC TCC TAG TTT CTT GAG GGA GAA A	58	150-300	ARMs PCR	GF112096
*(Xrgc950)**		R inner: AAA TCA ATC TGA TTG ATG AAT TCG C				GF112088
		F outer: GCT TGT TTA GTT TGA TGC TCC ATT AGA				
		R outer: ACA AGT TTA AAT TTG CCA ACA TGT TGT				
c82	8	F inner: GTT CAG GTA GAA TAC ATG AGA ACT AAA ATC	56	75-110	ARMs PCR	GF112097
*(Xrgc82)**		R inner: ATA GTT GCA TGT TGC CAA GTT TAA CA				GF112089
		F outer: ACT AGC TTC CTT TTA GGA GAC ATT GA				
		R outer: ACT GTA GAT GTT TTG ATG ACA AAT GAA				
r1534	8	F inner: TGA TCT GCT TAA GTG CAT TTA GCT ATC	56	100-200	ARMs PCR	GF112098
*(Xrgr1534.2)**		R inner: ACT GTG CTT TGA TGA AGA TAC TAC GAA				GF112090
		F outer: TCC AAT TGA TTG AAT TGA TTA ACA GTT				
		R outer: AAA AAA GGT ACA GAG CTT TTT TTG CTA				
c389-1	8	F Sv_allele: GTA CCA TGT ATG TTT TAC TTT TCG	56	100-300	2% Agarose gel	GF112099
*(Xrgc389-1)**		R Sv_allele: GCA GTC CAA TGT CGG TGA C				GF112091

		F B100_allele: ATC CAC AGG GCT CTC AGG				
		R B100_allele: ACA TAC GTG CTA TCA GCA GAA TG				
c1361	9	F inner: ACA TAG CAG CAT GGA CAG GTG TA	54	100-180	ARMs PCR	GF112100
*(Xrgc1361)**		R inner: CAT TTA CTT CCT CTG TAC AAA AAT CGC				GF112092
		F outer: GAG TAA TTT CAA GCA TTC TTT GCT CTT				
		R outer: TTG TCT CAG GGA AGA TAG CTT TGT ATT				
TEOSINTE BRANCHED	9		59	200-700	PCR amplicons	GF112101
1 (TB1)					digested with	GF112093
					DdeI at 37 °C	

Locus, linkage group, primer combinations, reaction conditions (where different from general description in text), polymorpism detection method, and Genbank accession number for STS markers (first number in each case is for *S. italica* B100 and the second is for *S. viridis* A10). An asterisk (*) after the locus name denotes an RFLP marker (WANG *et al.* 1998).

Table S2 Differences between the parents of the cross in individual trials.

Trial	*S. viridis* A10 days to flowering	*S. italica* B100 days to flowering	Difference	Additive effect from RILs	Additive effect as % parental difference
GH1-OK	20.2	39.8	19.7	10.3	52.4
GH2-OK	22.9	43.6	20.7	6.5	31.4
GC-BT	19.0	37.5	18.5	17.1	92.4
GC-OK	25.2	55.0	29.8	19.1	64.1
F1-OK	26.5	53.8	27.3	7.9	29.0
F2-OK	29.4	50.6	21.3	6.4	30.1
F1-GA	20.0	30.5	10.5	4.2	40
F2-GA	23.9	30.1	6.1	11.6	188.8

References

Gadberry, M. D., S. T. Malcomber, A. N. Doust and E. A. Kellogg, 2005 Primaclade - a flexible tool to find conserved PCR primers across multiple species. Bioinformatics 21: 1263-1264.

Giegerich, R., F. Meyer and C. Schleiermacher, 1996 GeneFisher--software support for the detection of postulated genes. Proc Int Conf Intell Syst Mol Biol 4: 68-77.

Gupta, S., K. Kumari, P. P. Sahu, S. Vidapu and M. Prasad, 2012 Sequence-based novel genomic microsatellite markers for robust genotyping purposes in foxtail millet *Setaria italica* (L.) P. Beauv. Plant Cell Reports 31: 323-337.

Jia, X. P., Y. S. Shi, Y. C. Song, G. Y. Wang, T. Y. Wang *et al.*, 2007 Development of EST-SSR in foxtail millet (*Setaria italica*). Genetic Resources and Crop Evolution 54: 233-236.

Jia, X. P., Z. H. Zhang, Y. H. Liu, C. W. Zhang, Y. S. Shi *et al.*, 2009 Development and genetic mapping of SSR markers in foxtail millet *Setaria italica* (L.) P. Beauv. Theoretical and Applied Genetics 118: 821-829.

Missiaggia, A., and D. Grattapaglia, 2006 Plant microsatellite genotyping with 4-color fluorescent detection using multiple-tailed primers. Genetics and Molecular Research 5: 72-78.

Schuelke, M., 2000 An economic method for the fluorescent labeling of PCR fragments. Nature Biotechnology 18: 233-234.

Wang, Z. M., K. M. Devos, C. J. Liu, R. Q. Wang and M. D. Gale, 1998 Construction of RFLP-based maps of foxtail millet, *Setaria italica* (L.) P. Beauv. Theoretical and Applied Genetics 96: 31-36.

Ye, S., S. Dhillon, X. Y. Ke, A. R. Collins and I. N. M. Day, 2001 An efficient procedure for genotyping single nucleotide polymorphisms. Nucleic Acids Research 29: art. no.-e88.
