# Supplementary material for: Genetic Control and Comparative Genomic Analysis of Flowering Time in Setaria (Poaceae)
Source: G3 (Bethesda). 2013 Feb 1;3(2):283–95. doi: 10.1534/g3.112.005207 (PMC3564988; doi:10.1534/g3.112.005207)
Supplement: Supporting Information [file supp_3.2.283_FigureS1.pdf]

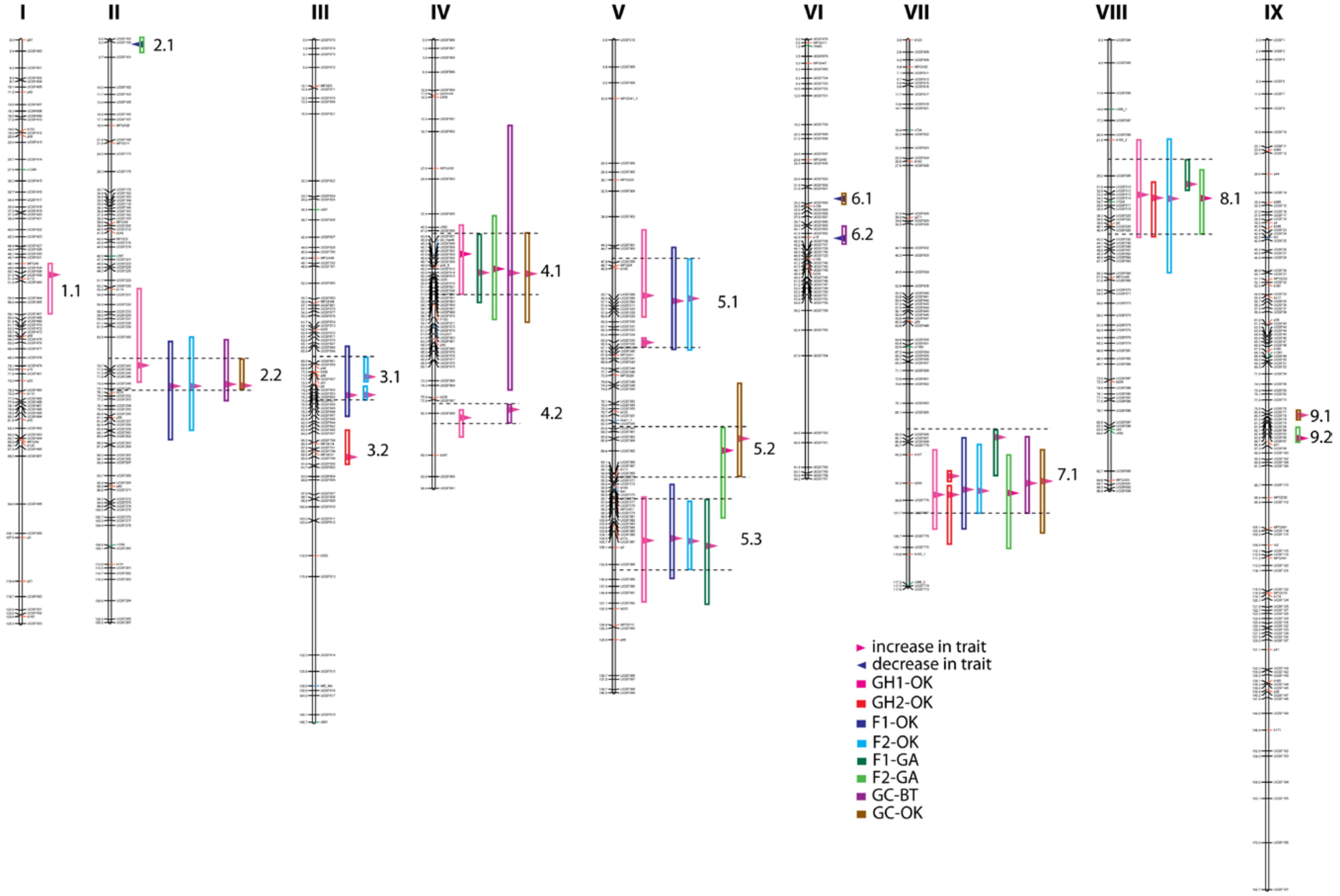

Figure S1 Full QTL map with all QTL found. Dashed black lines delimit common QTL regions defined by overlapping QTL intervals. Candidate genes were examined for common regions for QTL identified in multiple trials and for the entire interval for QTL found in a single trial. GH = Greenhouse, GC = Growth Chamber, F = Field. OK = Oklahoma State University, Stillwater, OK; BT = Boyce Thompson Institute, Ithaca, New York; GA = University of Georgia, Athens, GA.
