# Supplementary material for: Genetic Control and Comparative Genomic Analysis of Flowering Time in Setaria (Poaceae)
Source: G3 (Bethesda). 2013 Feb 1;3(2):283–95. doi: 10.1534/g3.112.005207 (PMC3564988; doi:10.1534/g3.112.005207)
Supplement: Supporting Information [file supp_3.2.283_FigureS2.pdf]

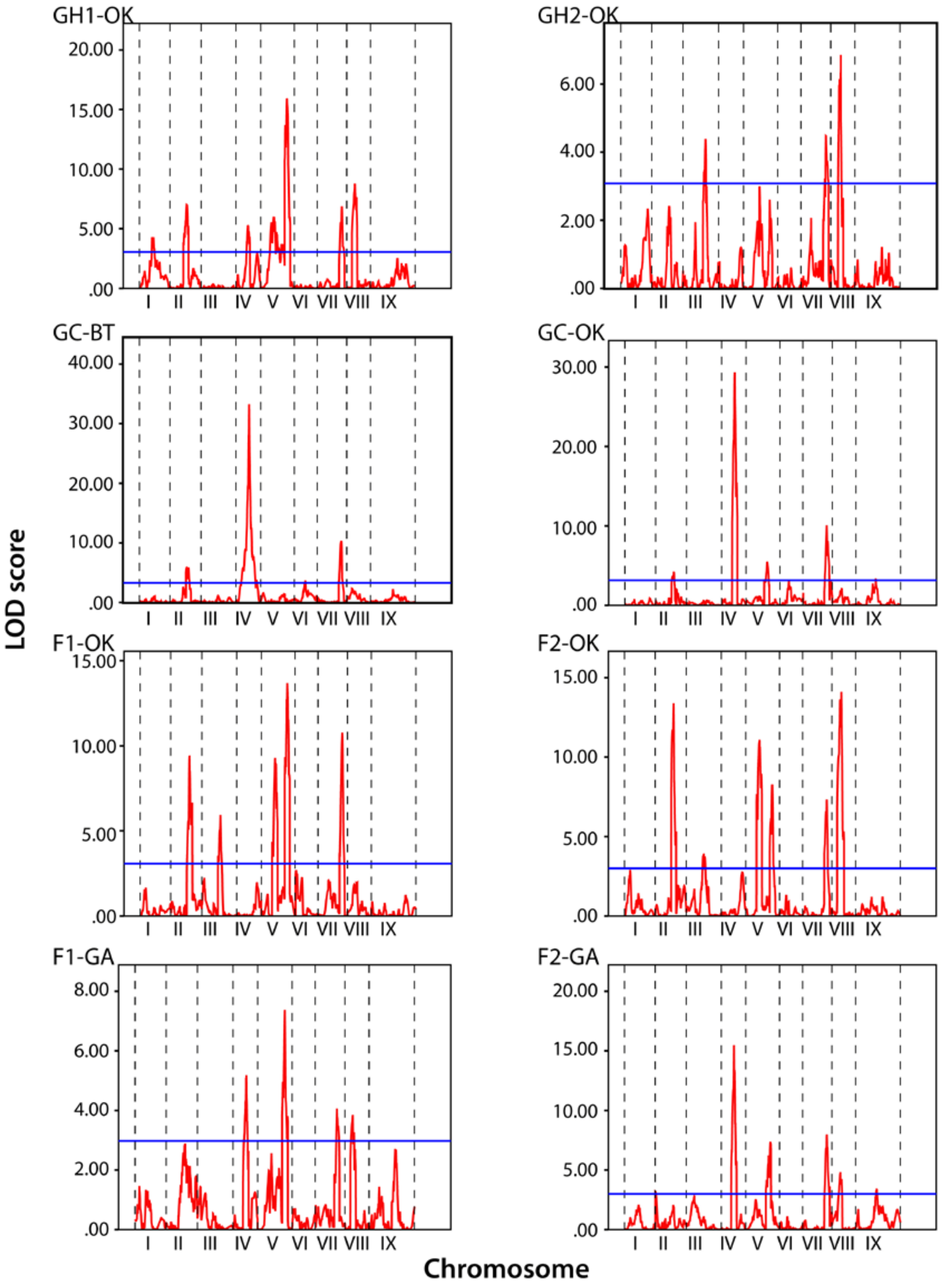

Figure S2 LOD graphs for each of the eight trials. Each graph shows the LOD scores for each trial along each chromosome in Setaria, with chromosomes lined up end to end (I-IX). The horizontal blue line represents the P < 0.05 significance level, so that QTL are declared significant if the peaks are above that line. In several cases there are multiple peaks underlying a single QTL. GH = Greenhouse, GC = Growth Chamber, F = Field. OK = Oklahoma State University, Stillwater, OK; BT = Boyce Thompson Institute, Ithaca, New York; GA = University of Georgia, Athens, GA.
