# Supplementary material for: Genetic Control and Comparative Genomic Analysis of Flowering Time in Setaria (Poaceae)
Source: G3 (Bethesda). 2013 Feb 1;3(2):283–95. doi: 10.1534/g3.112.005207 (PMC3564988; doi:10.1534/g3.112.005207)
Supplement: Supporting Information [file supp_3.2.283_TableS1.pdf]

Table S1 Primers sequences, detection method, and Genbank accession number for STS markers

Locus	Linkage group	Primer sequence (5′ →3′)	Ta °C	Fragment sizes (bp)	Polymorphism detection method	Genbank Accession Number
c1246	1	F inner: AAA GTG TCG TTT TAA TTA GTA CAG TTG TC	52	160-320	ARMs PCR	GF112102
*(Xrgc1246)**		R inner: TGA TTG CCA CAG AAC TAG TAT CTT TAT				GF112117
		F outer: GGA TAG ATG CTA TTC AGT AAG ACA CTA AA				
		R outer: TGC TTT TTC TTC AAC TAT GAT ATG TAT C				
c397	2	F inner: TTA TGT CTG CAA GTG ATC AAT TGT GAG TT	52	100-200	ARMs PCR	GF112103
*(Xrgc397)**		R inner: AAA TTC TGC ATG TAA AAT GAG TCC CG				GF112118
		F outer: GTG ATT CAC CTT CTC AGC TTA AGG CAT				
		R outer: GTA TTC CTG TGC AAT CTG TCT TAC TGC A				
r1789	2	F: TCG TTG GCG TGC CAC TGA AA	58	308	PCR amplicons	GF112104
*(Xrgr1789)**		R: TGG TCC TGC ACT CAG AAG CAC A			digested with	GF112119
					BsmF1 at 65 °C	
c901	3	F inner: ACT CCC ATT CTC TCG CGT TCC TTC G	64	80-140	ARMs PCR	GF112105
*(Xrgc901)**		R inner: CTG ACC CCT GGA GGC TGG GGT				GF112120
		F outer: AAG GAG GCT GGT GTC ACC TAT CAC CG				
		R outer: CCC TGT GCT AGA CAG GAT AGA CGG GGA				
BARREN	3	F: GGT ACG TGC AGG TTC TAC GC	56	254-257	3730 Fragment	GF112106
INFLORESCENCE 2 -		R: TTC AAC CGG AAC AGG TGG T			analysis	GF112121
LIKE (bif2-like)					DMSO	
c597	3	F inner: TAG CAG AGA TGA AAA GGT ATA GAA CAG CG	52	130-260	ARMs PCR	GF112107
*(Xrgc597)**		R inner: CTT TTC AAC GAC AAG ATT CAG GTA CGT				GF112122
		F outer: ATG CTT GAC AGG TAT ACC AGT GCA AG				

		R outer: TCT GTA TAA CAC TTG CAG GAA GGA CG				
c562	4	F inner: ACG CCA GGG ATT ATG TGA CTT TTT AG	60	130-250	ARMs PCR	GF112108
*(Xrgc562)**		R inner: TTC CTG CAA GAG TTT CAA TAT GAG GAT				GF112123
		F outer: CAG ATA GGA AGT AGC CAT TAA TGC CTG				
		R outer: AAG CTT GGT ATG AGA AAC GAG TGT GTC				
DWARF3 (D3)	4	F: GTC CCA CAG CGG GCA GTG TC	61	75-430	PCR amplicons	GF112109
		R: CGG CAT CCA CCA GCC AAG GG			digested with	GF112124
					HaeIII at 37 °C	
c235	4	F inner: GTT TCG ACT GCA AAA TTA TGA AAC TAC C	56	50-200	ARMs PCR	GF112110
*(Xrgc235)**		R inner: AGT TCT CAC TTC TCA AGT CCC AAA CA				GF112125
		F outer: GAA ATG AGA ACT ACC ATT CAA CAT TCG				
		R outer: AGA AAA ACC AAT TTC ATC AAA AGA ACG				
MONOCULM 1 (MOC1)	4	F inner: CCT CGA GCG CCT CGA ACA CCT CC	66	50-180	ARMs PCR	GF112111
		R inner: CCG TCG CCA TGG ACC ACT ACG CA			DMSO	GF112126
		F outer: AGC ACC TCC TGC TCC ACC GCC AG				
		R outer: GTG ACC GTC GCG GAG AGG GAG ATG AT				
BARREN STALK 1 (BA1)	5	F: CCA TCT TTC TTT GAT CCC TGT C	56	225-240	3730 Fragment	GF112112
		R: GGG CAG ATA TGG TCA TTT CAC			analysis	GF112127
MORE AXILLARY	5	F inner: GTC GGC TTT CTG GAC AGG CCG CAA	66	70-220	ARMs PCR	GF112113
BRANCHES 1 (MAX1)		R inner: CAG GCG GCA TTC GGC GCG TAC			DMSO	GF112128
		F outer: GTC ATC CCT GGC GTG GCT CTT CCC AT				
		R outer: CCA CCT TGC CGG ATT GAT TCC AAC CAT				
r1943	6	F inner: CAT CAT TCA CTG GTC CTA TAC ATG AG	56	150-300	ARMs PCR	GF112114
*(Xrgr1943)**		R inner: GAA ATG AGT CTG GAA GAA CAT GAA G				GF112129
		F outer: CGA ATA TAT AAC CCA CAA GAT GAA AAG				
		R outer: TAG CAA TAT TCA TTT TTA TTG GCA ATT				

c389_2	7	F: GAT CCC ATT GGT CTT GCA ACT	56	310-330	3% Agarose gel	GF112115
*(Xrgc389)**		R: ACA AGG TTC CAC CTC AAC CTG				GF112130
c1100	7	F: ATG AAT GCG GTG CTC TGT G	56	169-175	3% Agarose gel	GF112116
*(Xrgc1100)**		R: GGA AAA AGG GTG ACC TTC CT				GF112131
g271	7	F inner: GTC ATC GCC TAC AAC CCC ACA	59	75-190	ARMs PCR	GF112094
*(Xrgg271)**		R inner: TTG TCT TCT TCG GAC AGG GGG				GF112086
		F outer: GAC CTA CTG GTG GAA CAG CCA GAA				
		R outer: ACA AGA TGA GCA CGG AGT ATC ACT GAT				
c734	7	F inner: CTA TGC AAA GCT TTA TGT ATG CGA A	54	100-180	ARMs PCR	GF112095
*(Xrgc734)**		R inner: CCT CTA TAT ATG AAC AAC TTA ATT CTG				GF112087
		F outer: ATT TAG TCT GTA GTT ACA CCT ACT TCT GTG				
		R outer: ACA GAT ACC ATG AAG ATT AAA GAG TCA				
c950	8	F inner: TCC TCC TAG TTT CTT GAG GGA GAA A	58	150-300	ARMs PCR	GF112096
*(Xrgc950)**		R inner: AAA TCA ATC TGA TTG ATG AAT TCG C				GF112088
		F outer: GCT TGT TTA GTT TGA TGC TCC ATT AGA				
		R outer: ACA AGT TTA AAT TTG CCA ACA TGT TGT				
c82	8	F inner: GTT CAG GTA GAA TAC ATG AGA ACT AAA ATC	56	75-110	ARMs PCR	GF112097
*(Xrgc82)**		R inner: ATA GTT GCA TGT TGC CAA GTT TAA CA				GF112089
		F outer: ACT AGC TTC CTT TTA GGA GAC ATT GA				
		R outer: ACT GTA GAT GTT TTG ATG ACA AAT GAA				
r1534	8	F inner: TGA TCT GCT TAA GTG CAT TTA GCT ATC	56	100-200	ARMs PCR	GF112098
*(Xrgr1534.2)**		R inner: ACT GTG CTT TGA TGA AGA TAC TAC GAA				GF112090
		F outer: TCC AAT TGA TTG AAT TGA TTA ACA GTT				
		R outer: AAA AAA GGT ACA GAG CTT TTT TTG CTA				
c389-1	8	F Sv_allele: GTA CCA TGT ATG TTT TAC TTT TCG	56	100-300	2% Agarose gel	GF112099
*(Xrgc389-1)**		R Sv_allele: GCA GTC CAA TGT CGG TGA C				GF112091

		F B100_allele: ATC CAC AGG GCT CTC AGG<br>R B100_allele: ACA TAC GTG CTA TCA GCA GAA TG				
c1361<br>*(Xrgc1361)**	9	F inner: ACA TAG CAG CAT GGA CAG GTG TA<br>R inner: CAT TTA CTT CCT CTG TAC AAA AAT CGC<br>F outer: GAG TAA TTT CAA GCA TTC TTT GCT CTT<br>R outer: TTG TCT CAG GGA AGA TAG CTT TGT ATT	54	100-180	ARMs PCR	GF112100<br>GF112092
TEOSINTE BRANCHED 1 (TB1)	9		59	200-700	PCR amplicons digested with DdeI at 37 °C	GF112101<br>GF112093

Locus, linkage group, primer combinations, reaction conditions (where different from general description in text), polymorpism detection method, and Genbank accession number for STS markers (first number in each case is for *S. italica* B100 and the second is for *S. viridis* A10). An asterisk (*) after the locus name denotes an RFLP marker (WANG *et al.* 1998).
