# Supplementary material for: Genetic Control and Comparative Genomic Analysis of Flowering Time in Setaria (Poaceae)
Source: G3 (Bethesda). 2013 Feb 1;3(2):283–95. doi: 10.1534/g3.112.005207 (PMC3564988; doi:10.1534/g3.112.005207)
Supplement: Supporting Information [file supp_3.2.283_TableS2.pdf]

Table S2 Differences between the parents of the cross in individual trials.

Trial	*S. viridis* A10 days to flowering	*S. italica* B100 days to flowering	Difference	Additive effect from RILs	Additive effect as % parental difference
GH1-OK	20.2	39.8	19.7	10.3	52.4
GH2-OK	22.9	43.6	20.7	6.5	31.4
GC-BT	19.0	37.5	18.5	17.1	92.4
GC-OK	25.2	55.0	29.8	19.1	64.1
F1-OK	26.5	53.8	27.3	7.9	29.0
F2-OK	29.4	50.6	21.3	6.4	30.1
F1-GA	20.0	30.5	10.5	4.2	40
F2-GA	23.9	30.1	6.1	11.6	188.8

References

Gadberry, M. D., S. T. Malcomber, A. N. Doust and E. A. Kellogg, 2005 Primaclade - a flexible tool to find conserved PCR primers across multiple species. Bioinformatics 21: 1263-1264.

Giegerich, R., F. Meyer and C. Schleiermacher, 1996 GeneFisher--software support for the detection of postulated genes. Proc Int Conf Intell Syst Mol Biol 4: 68-77.

Gupta, S., K. Kumari, P. P. Sahu, S. Vidapu and M. Prasad, 2012 Sequence-based novel genomic microsatellite markers for robust genotyping purposes in foxtail millet *Setaria italica* (L.) P. Beauv. Plant Cell Reports 31: 323-337.

Jia, X. P., Y. S. Shi, Y. C. Song, G. Y. Wang, T. Y. Wang *et al.*, 2007 Development of EST-SSR in foxtail millet (*Setaria italica*). Genetic Resources and Crop Evolution 54: 233-236.

Jia, X. P., Z. H. Zhang, Y. H. Liu, C. W. Zhang, Y. S. Shi *et al.*, 2009 Development and genetic mapping of SSR markers in foxtail millet *Setaria italica* (L.) P. Beauv. Theoretical and Applied Genetics 118: 821-829.

Missiaggia, A., and D. Grattapaglia, 2006 Plant microsatellite genotyping with 4-color fluorescent detection using multiple-tailed primers. Genetics and Molecular Research 5: 72-78.

Schuelke, M., 2000 An economic method for the fluorescent labeling of PCR fragments. Nature Biotechnology 18: 233-234.

Wang, Z. M., K. M. Devos, C. J. Liu, R. Q. Wang and M. D. Gale, 1998 Construction of RFLP-based maps of foxtail millet, *Setaria italica* (L.) P. Beauv. Theoretical and Applied Genetics 96: 31-36.

Ye, S., S. Dhillon, X. Y. Ke, A. R. Collins and I. N. M. Day, 2001 An efficient procedure for genotyping single nucleotide polymorphisms. Nucleic Acids Research 29: art. no.-e88.
