# Supplementary material for: Genetic Control and Comparative Genomic Analysis of Flowering Time in Setaria (Poaceae)
Source: G3 (Bethesda). 2013 Feb 1;3(2):283–95. doi: 10.1534/g3.112.005207 (PMC3564988; doi:10.1534/g3.112.005207)
Supplement: Supporting Information [file supp_3.2.283_FileS1.pdf]

File S1

Supporting Information

1. Plant materials, experimental design and growing conditions

Greenhouse Trials: The first greenhouse trial (GH1-OK) was grown in 2008 in a greenhouse without additional lighting at Oklahoma State University during the months of June to September. Day length varied between 13.8 and 14.3 hours, with an average daily temperature of 26 °C, and light intensity of 1400 µmol $cm^{-2}s^{-1}$. The experimental design consisted of a Randomized Complete Block Design (RCBD) with six blocks and one replicate of each RIL per block. RILs were planted in 11x11x11 cm square pots (approximately 1210 cm^3), filled with Metro-Mix 366 (Sun Gro Horticulture Canada Ltd.) and spaced 15.5 cm apart. Plants were irrigated as needed with an aqueous complete fertilizer mix (Jack's mix: Nitrogen, Phosphorous and Potassium - 20:20:20).

The second greenhouse trial (GH2-OK) was grown in 2010 with additional metal halide and high-pressure sodium lighting at Oklahoma State University during the months of July to September. Day length, consisting of natural light with supplemental lighting before dawn and after dusk, was 16 hours, with an average daily temperature of 26.5 °C, and light intensity of 1400 µmol $cm^{-2}s^{-1}$. In this trial only 107 RILs were evaluated due to limitations in space. The experimental design consisted of a RCBD with four blocks, two planted at high density (11 cm apart) and two at low density (31 cm apart), with one replicate of each RIL per block. RILs were planted in 11x11x14 cm square pots (approximately 1573 cm^3), filled with Metro-Mix 366 (Sun Gro Horticulture Canada Ltd.). Plants were irrigated as needed with an aqueous complete fertilizer mix (Jack's mix: Nitrogen, Phosphorous and Potassium (20-20-20)).

Field Trials: Two trials were conducted at the Cimarron Valley Field Research Station in Perkins, Oklahoma during the months of May to July of 2010 and 2011. Seed was germinated in a greenhouse and transplanted to the field two weeks after sowing, at approximately the two-leaf stage. Day length varied between 14 and 14.3 hours, with an average daily temperature of 26.5˚C in 2010 and 28˚C in 2011. The first of these Oklahoma field trials (F1-OK) consisted of a RCBD with two blocks, with three replicates of each RIL per block. RILs were planted 25 cm apart from each other in rows. In order to mitigate the variable competition effects stemming from RILs of different sizes growing together, RIL rows were separated by rows of border plants, with rows 25cm apart. A commercial foxtail millet variety (German millet) was used for the borders and plants were watered as needed. One nitrogenous fertilizer application (as urea) was done in the sixth week of growth at a rate of 50 lb/acre (56 kg/ha). The second trial (F2-OK) was also a RCBD with four blocks, two planted at high density (15 cm apart) and two at low density (60 cm apart), with four replicates of each RIL per block. German millet was used as border plants, as in the first field trial. Blocks were pretreated with nitrogenous fertilizer (as urea) at a rate of 50 lb/acre (56 kg/ha), and with pre-emergent herbicide (Prowl® H2O herbicide, BASF Corp.).

Two field trials were also conducted at the University of Georgia, Athens, in June-July 2011 and July-August 2011 (14.3 hours day length, average temperature of 28 ^{0}C). These trials are labeled F1-GA and F2-GA. For the first trial (F1-GA), seeds were sown in soil filled peat pots (1 seed/pot). The pots were placed at 4 ^{0}C for 1 week after which they were placed in the glasshouse under natural light conditions. Twenty days later, the plants were planted in the field in hill plots in a RCBD with two blocks. For the second trial, ~100 seeds per RIL were sown in soil in pots, placed at 4 ^{0}C for 4 days and then in the glasshouse under 14.2 hours natural day light for four days. Two-day old seedlings were transplanted to soil-filled peat pots (1 plant/pot), and 10-day old seedlings were planted in hill plots in

a RCBD with two blocks. In both trials, hill plots were separated by 36" and each hill plot contained a single RIL in four replicates. Plots were irrigated when necessary.

Growth Chamber Trials: The first growth chamber experiment (GC-BT) was conducted at the Boyce Thompson Institute. Plants were grown under artificial light with 12 hours of light (750 μmol.m^{-2}.s^{-1}) and 12 hours of darkness. Average temperature was 31˚C during the light period and 25˚C during the dark. Five plants per RIL were planted in 220.5 cm^{3} pots filled with metro mix 360 (Sun Gro Horticulture), and thinned to two plants 10 days after sowing. Fertilization was applied every 7 days during the experiment (N-P-K; 20-20-20).

The second growth chamber experiment (GC-OK) was conducted in 2012 at Oklahoma State University. Plants were grown under artificial light with 12 hours of light (350 μmol.m^{-2}.s^{-1}) and 12 hours of darkness. Average temperature was 28˚C during the light period and 22˚C during the dark. Only 126 RILs were evaluated in this experiment due to space limitations, with RILs chosen to represent the extremes of flowering time (data taken from previous greenhouse experiments). The experiment had a single block with three replications per RIL, with plants spaced 15 cm apart. RILs were planted in 11x11x14 cm square pots (approximately 1573 cm^{3}), filled with Metro-Mix 366 (Sun Gro Horticulture Canada Ltd.). Plants were irrigated as needed with an aqueous complete fertilizer mix (Jack's mix: Nitrogen, Phosphorous and Potassium (20-20-20)).

Parental trial under different photoperiods: *Setaria italica* and *S. viridis* plants were grown in growth chambers at 28˚C day and 22˚C night temperatures. The long day photoperiod regime had 16 hours light while the short day regime had 12 hours light (350 μmol.m^{-2}.s^{-1}). Number of leaves on the culm, average speed of leaf initiation, time taken until inflorescence meristem starts to initiate, and time of inflorescence emergence were measured for each species in each environment. Plants were grown in 11x11x14 cm square pots (approximately 1573 cm^{3}), filled with Metro-Mix 366 (Sun Gro Horticulture Canada Ltd.), and irrigated as needed with an aqueous complete fertilizer mix (Jack's mix: Nitrogen, Phosphorous and Potassium (20-20-20)).

2. Molecular Marker Development and Genotyping

Almost two hundred published SSR primer pairs (Jia *et al.* 2007; Jia *et al.* 2009; Gupta *et al.* 2012) were tested on the parents and a total of 126 informative markers were chosen to genotype the RIL population. PCR fragment separation for SSR markers was done via agarose gels (1 to 3 % depending on fragment sizes) or with an ABI PRISM 3730 Genetic Analyser (Applied Biosystems). Amplification reactions for SSR fragments separated in agarose gels were done in a 10 µl volume containing 0.5 Units of Taq DNA polymerase (Promega Corp), 1.5 mM $MgCl_2$, 100 µM dNTPs, 20 ng of genomic DNA, 2.0 µl of 5x GoTaq buffer (Promega Corp) and 0.32 µM of forward and reverse primer. Some primers required the addition of 5% DMSO (indicated DMSO in Supplementary Table1) to consistently amplify. PCR cycling conditions consisted of an initial denaturation step at 94 °C for 2 min followed by 35 amplification cycles with an initial touchdown, and a final 5 min extension cycle at 72 °C. Each of the 35 cycles had a denaturation step at 94 °C for 40 s followed by 40s at the annealing temperature which was started 4 °C above the primers' melting temperature and decreased every two cycles by 2 °C to reach the primers' annealing temperature by the fifth cycle, and an extension step of 1 min at 72 °C. PCR fragments were visualized in 1 to 3 % agarose gels stained with ethidium bromide. Amplification reactions for SSR markers analyzed with the ABI PRISM 3730 Genetic Analyser (Applied Biosystems) were done using the "economic method" developed by Schuelke (Schuelke 2000) and further improved by Missiaggia and Grattapaglia (Missiaggia and Grattapaglia 2006). PCR reactions were done essentially as described

above, except that primer concentrations were 0.10 µM for the forward tailed primer, 0.3 µM for the reverse primer and 0.2 µM for the fluorescently labeled forward primer

To develop sequence-tagged-site (STS) markers for selected rice RFLP probes/sequences used in the F_2 foxtail genetic map (WANG *et al.* 1998) and for genes of interest (Supplementary Table 1), we first designed minimally degenerate primers to consensus regions of the rice sequences and their homologs in maize, sorghum and sugarcane. Sequences were obtained from Gramene (http://www.gramene.org) and the NCBI website (NCBI http://www.ncbi.nlm.nih.gov/gene), and aligned using Sequencher version 4.9 (Gene Codes Corporation) or MacClade version 4.0 (Maddison and Maddison 2000). Primers were designed with the programs GeneFisher (http://bibiserv.techfak.uni-bielefeld.de/genefisher2/) (GIEGERICH *et al.* 1996) and Primaclade (http://primaclade.org) (GADBERRY *et al.* 2005), and tested for amplification in the parental genotypes *S. italica* accession B100 and *S. viridis* accession A10. Single band PCR products were TOPO TA cloned (Invitrogen CA), and sequencing was done following the standard protocol for BigDye Terminator v1.1 (Applied Biosystems). Sequence reads were analyzed with Sequencher 4.9 and SNPs or fragment length polymorphisms were identified between the two parental genotypes. These were detected using two primer pairs to amplify, respectively, the two different alleles of a SNP in the same PCR reaction, incorporating mismatches that increase specificity in the 3' end of the primers that end on the SNP polymorphism (tetraprime ARMS-PCR) (YE *et al.* 2001). Primers for the ARMS-PCR technique were designed with the program Primer1 available at http://cedar.genetics.soton.ac.uk/public_html/primer1.html (Supplementary Table 1). PCR reactions for the ARMS-PCR technique were done in a 10 µl final volume. Reagent concentrations were the same as those for the SSR amplifications, except for primer concentrations that were 0.84 µM for each of the two inner primers and 0.084 µM for each of the two outer primers. Some primer pairs required the addition of 5% DMSO (indicated DMSO in Supplementary Table1). PCR cycling conditions were the same as those used for SSR amplifications. PCR fragments were visualized in 2% agarose gels stained with ethidium bromide.
